# Increasing flood hazard in the Lower Mississippi River due to extreme storm clustering

**DOI:** 10.1126/sciadv.adt1868

**Published:** 2025-10-01

**Authors:** Yuan Liu, Daniel B. Wright, Felipe Quintero, Alexander Michalek, Gabriele Villarini, James A. Smith

**Affiliations:** ^1^ Department of Civil and Environmental Engineering, University of Wisconsin-Madison, Madison, WI, USA.; ^2^ IIHR Hydroscience & Engineering, Iowa Flood Center, University of Iowa, Iowa City, IA, USA.; ^3^ Department of Civil and Environmental Engineering, Princeton University, Princeton, NJ, USA.

## Abstract

Although major floods in the Lower Mississippi River Basin (LMRB) are primarily driven by clusters of extreme storms rather than isolated events, the role of storm clustering in LMRB flood hazard remains underexplored. We show that floods driven by compound space-time storm clustering have higher peaks, volumes, and durations compared to those from isolated storms or single-mode clustering. Under future climate conditions (2070 to 2100; SSP3-7.0 scenario), projections indicate a 26% rise in the number of extreme storms, an 8% increase in average storm precipitation, and a 17% reduction in dry intervals preceding flood peaks. The dominant flood type is projected to shift from those produced by isolated storms (38%) to ones produced by compound storm clustering (49%)—the type of floods that have been responsible for catastrophic floods historically. These findings highlight the need to incorporate storm clustering and its changes into design flood analysis and management strategies for the LMRB.

## Teaser

Climate change intensifies extreme storm clusters and raises flood risks in the Lower Mississippi River.

## INTRODUCTION

**Fig. 1.**
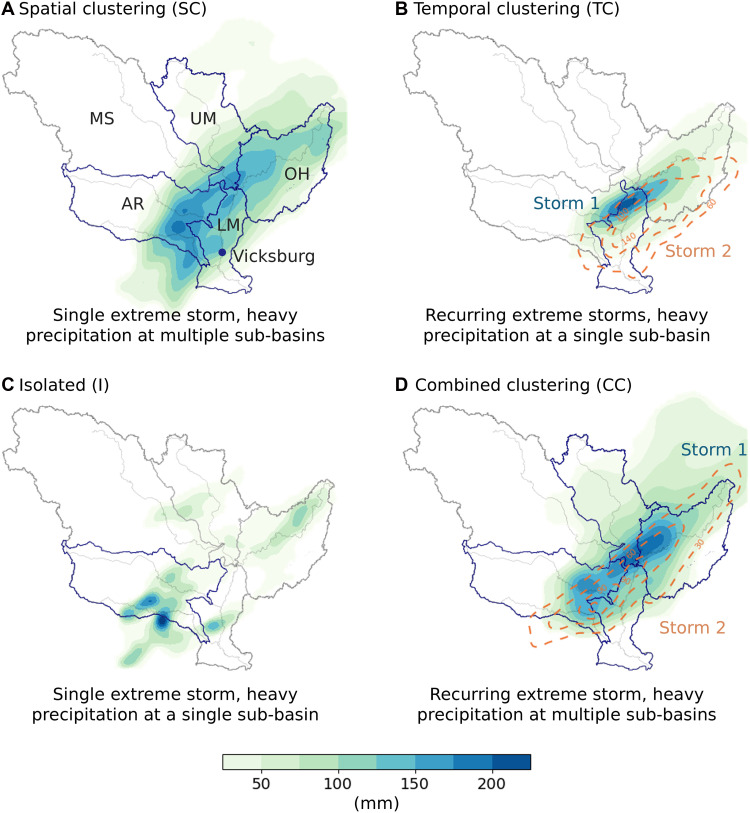
Typical storm clustering patterns preceding winter-spring flood peaks in the LMRB. (**A**) SC: A single extreme storm delivers heavy precipitation across multiple sub-basins (rainfall map from the March 2018 flood). (**B**) TC: Recurring storm events produce heavy precipitation in the same sub-basin (rainfall map from the March 2019 flood). (**C**) I: A single storm generates heavy precipitation in one sub-basin (rainfall map from the April 2020 flood). (**D**) CC: Multiple extreme storms, each delivering heavy precipitation across multiple sub-basins (rainfall map from the May 2011 flood). Shading and dashed contours indicate the first and second storms, respectively. Sub-basins affected by heavy precipitation are outlined in dark blue. The five major sub-basins of the MRB are as follows: Lower Mississippi (LM; 0.27 million km^2^), Ohio-Tennessee (OH; 0.52 million km^2^), Upper Mississippi (UM; 0.49 million km^2^), Missouri (MS; 1.3 million km^2^), and Arkansas-Red (AR; 0.64 million km^2^). The entire MRB is ~3.2 million km^2^.

Floods are among the costliest and most deadly disasters in the world, causing billions of dollars of economic damage and thousands of fatalities each year (*1*–*3*). The Lower Mississippi River Basin (LMRB; Fig. 1 and fig. S1), with a drainage area covering 40% of the contiguous United States, has experienced some of the most devastating floods in history (*4*, *5*). Notable events include the spring 2011 flood, the largest discharge at Vicksburg since the 1927 Mississippi Flood, resulting in $3.2 billion (in 2011 dollars) in agricultural losses and infrastructure damage (*5*, *6*). The spring 2019 flood was the longest-lasting flood on record, with many locations in the basin remaining above flood stage for more than 150 days (*7*). To mitigate flood hazards, the Lower Mississippi River is regulated by a complex system of upstream flood control reservoirs, levees, and a series of spillways that can be opened to relieve river stage during flood events (*8*, *9*). However, with increasing population and development along the river, a failure of this flood management system during an extreme flood event could lead to severe economic and social impacts (*10*). Estimates suggest that a repeat of the 1927 Mississippi Flood could cause over $100 billion in economic losses, with substantial global financial impacts (*11*). Therefore, understanding and accurately assessing future flood hazard is critical for regional development and hazard mitigation in the LMRB.

Major floods in the LMRB often result from clusters of extreme storms during winter and spring time, typically associated with strong water vapor transport from the Gulf of Mexico (*4*, *5*, *12*–*15*). For instance, the spring 2011 flood was driven by two successive storm systems during 17 to 28 April and 29 April to 4 May, each delivering heavy precipitation across multiple sub-basins (*14*). Flood events are often also influenced by factors such as above-normal snowmelt (SM) in the Upper Mississippi and Missouri River Basins, as well as elevated river levels and saturated soil due to heavy rainfall in the preceding months (*9*, *16*). The design flood in the LMRB, known as “Hypo-Flood 58A,” was developed by combining three historical extreme storms from 1937, 1950, and 1938 into a 4-week-long sequence on the basis of meteorological reasoning (*12*). This hypothetical storm cluster provides a purported “worst-case” flood scenario along the Lower Mississippi River and has formed the basis for the design capacity of flood management infrastructure (*17*).

The clustering of extreme storms relates closely to spatial and temporal compounding hazards (*18*), which, however, have received limited attention in the Mississippi River Basin (MRB). Spatial compounding occurs where multiple connected locations experience the same or different hazards within a short time frame, amplifying the overall impact (*19*). In the context of LMRB floods, this involves a large storm system delivering heavy precipitation across several tributaries, triggering synchronized runoff generation and a combined flood wave in the Lower Mississippi River (*20*). Temporal compounding, on the other hand, involves successive hazards striking the same area, intensifying the outcome beyond that of a single event (*21*, *22*). In the LMRB, this often exhibits as multiple extreme storms producing recurring heavy rainfall in a single tributary over a short period (*15*). The initial storm saturates the soil and raises the baseflow, enabling subsequent storms to generate rapid surface runoff and amplified flood waves propagating to the main stem of the river.

The compounding impacts of extreme storm clusters have notable implications for flood hazard mitigation and recovery: Each storm in the cluster can deplete emergency resources, fill reservoirs, damage infrastructure, and heighten community vulnerability, making subsequent events more destructive as they strike a region still under recovery (*23*). This underscores an urgent need to understand and quantify extreme storm clustering patterns in the LMRB and their role in driving major floods. This task, however, is challenging due to data scarcity and quality issues for extremes—the worst compound events can be difficult to characterize given only 50 to 100 years of precipitation and river flow observations (*24*). The assessment becomes further complicated for future hazard scenarios as the intensity of storms and their spatiotemporal structures are projected to change under future climate scenarios (*18*, *25*–*27*).

This paper addresses these issues by modeling and analyzing LMRB floods driven by extreme storm clusters under historical and future climate scenarios. The results and discussion are presented in six sections. In the first section, we use StormLab, a stochastic rainfall generator, to simulate storm clustering scenarios over the MRB from 1901 to 2100, using large ensembles from four global climate models (GCMs). We simulate flood discharge in the LMRB with the Hillslope Link Model (HLM), a semidistributed hydrologic model, and examine the shifts in extreme storm clustering patterns preceding peak discharge under future climate conditions. The second section classifies simulated flood events into four types on the basis of storm clustering patterns, including isolated (I), spatial clustering (SC), temporal clustering (TC), and compound clustering (CC). We evaluate the characteristics and projected changes for each flood type and identify the dominant type that leads to the most severe floods.

In the third section, we use a linear model to assess the relative contributions of storm cluster precipitation, antecedent basin conditions, and SM to future peak discharge changes. The fourth section quantifies projected changes in extreme floods (e.g., 10,000-year events) driven by storm clustering, using a nonstationary generalized extreme value (GEV) model. We also estimate projected return periods for historical extreme floods and the design flood under future climate conditions. Sections five and six explore the implications of intensified storm clustering for the current flood management system and discuss key uncertainties and limitations in this study. We conclude by advocating for new rainfall and flood hazard analysis methods that account for spatial and temporal storm clustering rather than focusing on a single event. We also urge enhancements in weather forecasting and flood management strategies that incorporate storm clustering impacts to reduce increasing flood risks in the LMRB.

## RESULTS

### Extreme storm clusters before flood peaks

Historical observations indicate that major floods in the LMRB are primarily driven by clusters of extreme storms preceding flood peaks (e.g., Fig. 1 and figs. S2 to S4). To investigate this, we used a stochastic rainfall generator (StormLab) to simulate continuous precipitation fields at 6-hour and 0.03° resolution across the entire MRB (*28*). These simulations were conditioned on coarse-scale precipitation fields (6-hour, 1° resolution) obtained from four bias-corrected CMIP6 models: CESM2 (10 ensemble members), E3SM (10 ensemble members), MPI-ESM1-2-HR (10 ensemble members), and EC-Earth3 (8 ensemble members), yielding 38 ensemble members. Each ensemble spans historical (1901 to 2014) and projected future periods (2015 to 2100) under the SSP3-7.0 scenario (*29*). The resulting high-resolution precipitation fields, along with GCMs’ daily surface temperature and monthly evapotranspiration fields, were input to the HLM, a distributed hydrologic model, to simulate daily discharge responses along the Lower Mississippi River. We focused on peak events during winter and spring (December to May) at key gauge locations, which historically account for over 90% of peak flows (table S1).

We analyzed storm event characteristics within 30 days before winter-spring flood peaks at Vicksburg, a key flood monitoring location in the LRMB (see Fig. 1 and fig. S1), in both historical and future periods. The 30-day window was selected to capture the cumulative storm impacts that contribute to LMRB floods as historical floods are typically driven by storms that occurred within 30 days before flood peaks (*4*, *17*). Individual storm events were identified using a storm tracking algorithm (*30*), with storm characteristics derived from StormLab-simulated precipitation fields. Extreme storm events are defined here as those producing heavy precipitation (≥90th percentile of historical records) in at least one major sub-basin. Storm events that did not meet this criterion are referred to as nonextreme storms.

**Fig. 2.**
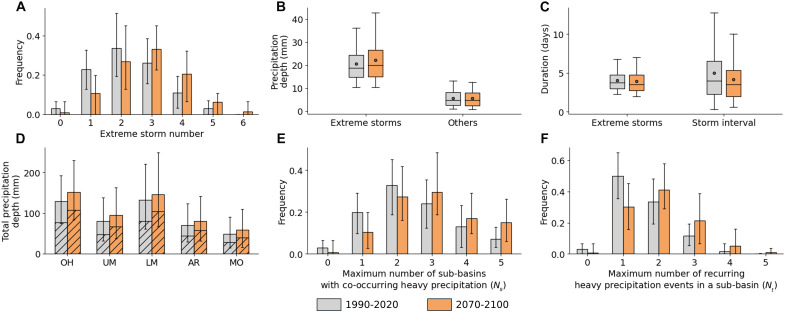
Characteristics of extreme storm clusters preceding winter-spring flood peaks at Vicksburg. (**A**) Number of extreme storms. Extreme storms are defined as events producing precipitation above the 90th percentile from historical records in at least one major sub-basin. (**B**) Precipitation depth of extreme storms and other nonextreme events. (**C**) Extreme storm duration and dry intervals. (**D**) Total precipitation depth in major sub-basins within 30 days before flood peaks. Hashed areas represent the average contribution from extreme storms. (**E**) Maximum number of sub-basins affected by heavy precipitation from a single storm event (*N_s_*). (**F**) Maximum number of recurring heavy precipitation events in the same sub-basin (*N_t_*). In (A) and (D) to (F), bars represent the mean. In (B) and (C), boxes span the first to third quartiles, with a line at the median and a dot at the mean. Whiskers represent the 5th to 95th percentiles.

Our analysis reveals notable shifts in extreme storm patterns. The average number of extreme storms preceding flood peaks increases by 26%, from 2.3 in 1990 to 2020 to 2.9 in 2070 to 2100 (Fig. 2A; Welch’s *t* value = −12.6; *P* < 0.001). The average precipitation depth of these extreme storms is projected to rise by 8% (Welch’s *t* value = −7.0; *P* < 0.001), whereas the depth of nonextreme storms shows a slight decline (3%; Welch’s *t* value = 2.5; *P* < 0.05). The average duration of extreme storms, historically 4.0 days in 1990 to 2020, remains relatively stable, with a slight 2% decrease by 2070 to 2100 (Fig. 2C; Welch’s *t* value = 2.2; *P* < 0.05). The average area of extreme storms is 730,000 km^2^ in 1990 to 2020, which is projected to increase slightly by 3% in 2070 to 2100 (Welch’s *t* value = −6.9; *P* < 0.001). In addition, the average interval between extreme storms shortens by 17%, dropping from 5.0 to 4.1 days (Fig. 2C; Welch’s *t* value = 5.1; *P* < 0.001). These changes in extreme storm frequency, intensity, area, and timing contribute to an increase in total 30-day precipitation before flood peaks across sub-basins, ranging from 21% in the LMRB to 26% in the Upper Mississippi River Basin (Fig. 2D).

We defined a storm as exhibiting strong SC if it produces heavy precipitation across multiple sub-basins. To quantify this, we introduced an SC metric *N_s_*, representing the maximum number of sub-basins experiencing heavy precipitation from a single storm event. To evaluate TC, we defined a TC metric *N_t_* as the number of consecutive storms causing heavy precipitation in the same sub-basin, with a dry interval of less than 7 days. A high *N_t_* value indicates strong TC, meaning multiple extreme storms affect the same sub-basin in rapid succession.

Our results show that the average *N_s_* increases from 2.5 in the historical period to 3.0 in the future period (a 20% rise; Fig. 2E; Welch’s *t* value = −10.0; *P* < 0.001), suggesting that a single storm is more likely to deliver heavy precipitation to multiple sub-basins. Similarly, the average *N_t_* rises from 1.6 to 2.0 (a 25% increase; Fig. 2F; Welch’s *t* value = −12.3; *P* < 0.001), indicating a greater tendency for recurring heavy precipitation events within the same sub-basin. The increases in *N_s_* and *N_t_* reflect enhanced SC and TC of extreme storms, amplifying runoff and discharge generation through interactions with the basin’s drainage network, which is explored in the next section.

### Flood characteristics under extreme storm clustering

The SC and TC of extreme storm events play a critical role in driving major floods in the LMRB. To explore this, we categorized simulated flood events into four types on the basis of storm clustering patterns preceding flood peaks: SC, TC, I, and CC. These types are defined as follows (see Fig. 1):

SC occurs when a single extreme storm delivers heavy precipitation across multiple sub-basins before the flood peak (Fig. 1A). The storm event produces synchronized runoff and discharge from major tributaries that converge into the mainstem, generating a large flood wave in the Lower Mississippi River. For example, the March 2018 flood resulted from a storm event between 13 and 26 February that produced heavy precipitation across four sub-basins (OH, LM, UM, and AR), yielding a peak discharge of 51,253 m^3^/s at Vicksburg (see Fig. 1A and fig. S2). We classified events as SC when the storm SC metric (*N_s_*) ≥ 3 and TC metric (*N_t_*) < 2, indicating heavy precipitation in at least three sub-basins and no recurring extreme storms in the same sub-basin.

TC involves recurring storm events that produce heavy precipitation in the same sub-basin (Fig. 1B). An initial storm saturates the soil, reducing its infiltration capacity; subsequent storms generate rapid surface runoff and a flood wave that quickly propagates to the main stem. This type can also produce cumulative stress on flood management infrastructure (e.g., dams, urban drainage systems, and levees) and heighten flash flood risks in the affected areas. A historical example is the March 2019 flood: The first storm (10 to 13 February) brought heavy precipitation to the LMRB, followed by a second storm (19 to 24 February) that again affected the LMRB and Ohio River Basin, resulting in a peak discharge of 53,235 m^3^/s at Vicksburg (see Fig. 1B and fig. S3). Classification as TC requires *N_s_* < 3 and *N_t_* ≥ 2, reflecting recurring extreme storms in a single sub-basin with limited SC.

I events involve a single storm producing heavy precipitation in only one or two sub-basins, whereas other regions experience weak or moderate precipitation (Fig. 1C). This type may cause localized flooding in affected areas, but other tributaries remain relatively calm. The LMRB typically experiences short-lived peaks as the main river absorbs the inflow without catastrophic flooding. For instance, the April 2020 flood was driven by a storm primarily affecting the Arkansas River Basin, resulting in a moderate peak discharge of 44,175 m^3^/s at Vicksburg (see Fig. 1C and fig. S4). Classification as type I requires *N_s_* < 3 and *N_t_* < 2, reflecting minimal SC and TC.

CC represents the case when multiple extreme storms strike in rapid succession, each delivering heavy precipitation across multiple sub-basins (Fig. 1D). This type combines both SC and TC, creating a compound effect that produces more severe flooding. Successive widespread storms can affect the river system from multiple directions, producing discharge in major tributaries that converge into compound flood waves in the LMRB over a short period. The May 2011 flood is an example of this type: A first storm (17 to 28 April) hit four sub-basins [Ohio-Tennessee (OH), Lower Mississippi (LM), Upper Mississippi (UM), and Arkansas-Red (AR)], followed by a second storm affecting the Ohio River Basin, Arkansas River Basin, and LMRB, producing a peak discharge of 65,411 m^3^/s at Vicksburg that surpassed the 1927 discharge record (see Fig. 1D and fig. S5). Classification as CC requires *N_s_* ≥ 3 and *N_t_* ≥ 2, indicating both widespread and recurring heavy precipitation.

**Fig. 3.**
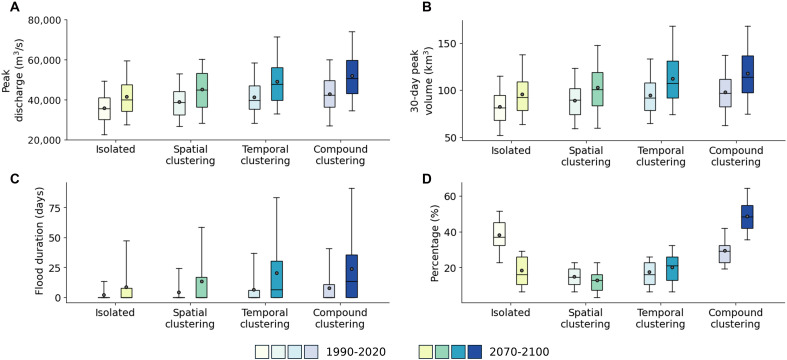
Characteristics of winter-spring flood events at Vicksburg across four flood types. (**A**) Winter-spring peak discharge. (**B**) Thirty-day peak volume. (**C**) Duration of flood hydrograph exceeding the major flood level defined by the US National Weather Service. (**D**) Relative portion of each flood type. Boxes span the first to third quartiles, with a line at the median and a dot at the mean. Whiskers represent the 5th to 95th percentiles. The color scale indicates different flood types, with transparent colors representing the historical climate period (1990 to 2020).

We examined key flood characteristics—peak discharge, 30-day peak volume, and flood duration—for each flood type in historical and future periods (Fig. 3). In the historical period, the CC type produced the highest mean peak discharge at Vicksburg (43,000 m^3^/s), which is 4, 10, and 20% higher than TC (41,000 m^3^/s), SC (39,000 m^3^/s), and I (36,000 m^3^/s), respectively (Fig. 3A). The CC type also exhibited the largest mean 30-day peak volume (98 km^3^) and flood duration (7.7 days), whereas the I type recorded the smallest (82 km^3^ and 2.1 days; Fig. 3B). The TC type fell slightly below CC (95 km^3^ and 6.6 days) but exceeded SC (89 km^3^ and 4.3 days). These results highlight that CC-type floods, driven by intense spatial and temporal storm clustering, typically generate the most severe flood conditions. The TC-type floods ranked the second, surpassing both SC and I types in magnitude.

In the projected future period, all flood types show increases in key flood characteristics. The CC type exhibits the greatest rise, with mean peak discharge increasing by 21% to 51,970 m^3^/s (Welch’s *t* value = −11.9; *P* < 0.001) and flood volume by 20% to 118 km^3^ (Welch’s *t* value = −11.3; *P* < 0.001), respectively. Its mean flood duration surges to 23.9 days with heightened variability (Welch’s *t* value = −10.7; *P* < 0.001). There is also a shift in the dominant flood type (Fig. 3D): In the historical period, the I type dominated (38%), followed by CC (30%), TC (17%), and SC (15%). In the future, the CC type dominates (49%), with TC rising to second in importance (20%). This suggests that the LMRB will experience much more frequent CC-type floods under future climate scenarios, characterized by amplified peak flows, flood volumes, and durations.

These changes carry notable implications for flood preparation and management. Traditional flood control measures, such as dam and spillway operations, are typically designed for single, localized storm events, but future strategies must adapt to address recurring heavy precipitation across multiple tributaries. These widespread, successive storms will place stress on tributary and downstream infrastructure, calling for structural upgrades and more frequent inspections and maintenance. In addition, weather and flood forecasting services should expand beyond predicting impending storms to include subsequent extreme events, enabling better-informed flood preparedness and response actions.

### Contributions of SM and antecedent conditions to peak discharge

In addition to extreme storm precipitation, we examined the contributions of basin antecedent conditions and SM to peak discharge under future climate scenarios. We defined the saturated area fraction (SAF) as the percentage of basin areas that have static tank storage ≥ 90% of maximum capacity on the 30th day before the flood peak (smoothed with a 7-day moving average). The static tank storage in the HLM reflects surface soil moisture of the hillslope, and high SAF values indicate saturated basin conditions. We also calculated the 30-day total precipitation (P_30_) and SM before flood peaks in the MRB, derived from precipitation simulations and HLM snow storage outputs. Results indicate that average P_30_ is projected to increase from 78 mm in 1990 to 2020 to 91 mm in 2070 to 2100, a 17% rise (Welch’s *t* value = −13.5; *P* < 0.001). The average SAF is 39% in 1990 to 2020, which is projected to increase to 42% in the future period (Welch’s *t* value = −10.0; *P* < 0.001). In contrast, SM decreases from 11 to 4 mm, showing a reduction of 60% (Welch’s *t* value = −20.2; *P* < 0.001).

A linear model was fitted to assess the relationships between peak discharge (*Q*) and the three factors (P_30_, SAF, and SM) using simulated flood events (*R*^2^ = 0.57, *P* < 0.001 for all factors). The contribution from each factor was quantified by predicting changes in *Q* on the basis of differences in their historical and future averages. Changes in P_30_ and SAF account for 70% [95% confidence interval (CI): 65 to 74%] and 57% (95% CI: 54 to 61%) of the projected change in peak discharge, respectively, whereas the decline in SM contributes −27% (95% CI: −33 to −20%). These findings highlight precipitation from extreme storm clusters as the primary driver of increased peak discharge in future climates. However, wetter antecedent basin conditions also play an important role, which is likely driven by increased December to May precipitation over the MRB (see fig. S10). Although the reduction in SM offsets flood peaks, its influence is notably smaller than that of P_30_ and SAF.

### Future likelihood of extreme floods

**Fig. 4.**
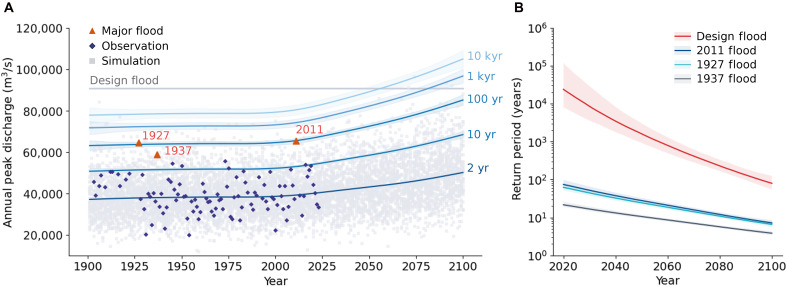
Changes in return levels of extreme winter-spring floods at Vicksburg. (**A**) Winter-spring peak discharge for return periods of 2 to 10,000 years (blue solid lines), with 95% prediction intervals (shaded areas). Purple diamonds represent peak discharge observations, with the three largest flood events marked by red triangles. Gray dots represent simulated peak discharges from 1901 to 2100. The gray horizontal line denotes the 1955 design flood peak discharge. kyr, thousand years; yr, years. (**B**) Return periods of flood peaks exceeding the design flood (red), 2011 (blue), 1927 (aqua), and 1937 (gray) Mississippi floods from 2020 to 2100, with 95% prediction intervals (shaded areas).

To assess changes in extreme flood frequency due to storm clustering, we fitted a nonstationary GEV model to simulated peak discharge data from 1901 and 2100. The GEV shape parameter was held constant, whereas the location and scale parameters varied as functions of time (expressed in years). Results reveal an increasing trend in extreme peak discharges at Vicksburg after 2020, with the 10,000-year flood projected to rise by 28% by 2100 relative to the 2020 baseline (Fig. 4A). Comparable trends were observed at other gauge stations (Arkansas City, Helena, and Memphis; see figs. S6 and S7).

The largest recorded flood peak at Vicksburg from the May 2011 flood (65,411 m^3^/s) had an estimated return period of 112 years (95% CI: 84 to 153 years) in 2011 based on the GEV model (Fig. 4B). Flood events of the same magnitude are projected to become far more frequent, with return periods decreasing to 23 years (95% CI: 20 to 27) by 2050 and 8 years (95% CI: 7 to 9) by 2100. The second- and third-largest historical peaks, from 1927 and 1937, had return periods of 116 years (95% CI: 91 to 156) and 33 years (95% CI: 28 to 39) in their respective years; by 2100, these are expected to shorten to 7 years (95% CI: 6 to 8) and 4 years (95% CI: 3 to 4), respectively. These findings suggest that historically catastrophic floods will occur more frequently under future climate scenarios, driven by intensified extreme storm clusters.

The 1955 design flood study estimated a “worst case” peak discharge of 83,817 m^3^/s at Vicksburg under “unregulated” conditions, i.e., without upstream reservoirs or flood control structures (*17*). In 2020, this design flood had an equivalent return period of 57,500 years (95% CI: 15,600 to 569,000 years), but this is projected to drop markedly to 1700 years (95% CI: 900 to 3700 years) by 2050 and 150 years (95% CI: 90 to 250 years) by 2100. This indicates a substantial decline in the reliability of the existing flood management system, which was designed to contain flows at the design flood level.

### Flood management in the LMRB

Flood control structures and upstream reservoirs play a crucial role in reducing extreme floods in the LMRB. Before 1927, flood control over the basin relied solely on levees, which failed catastrophically during the 1927 flood (*31*). The 1928 Flood Control Act introduced basin-wide mitigation measures, including reinforced levees, floodways, channel stabilization, and upstream reservoirs (*32*). The reservoir systems built along the mainstems and tributaries in the major sub-basins provide storage capacity to reduce flood peak magnitude. The Old River Control Structure, located downstream of the Lower Mississippi River (fig. S1), maintains a flow split of ~70% for the Mississippi River and 30% for the Atchafalaya River to stabilize the river channel during extreme floods (*9*). In addition, the Morganza Floodway and Bonnet Carré Spillway can be operated in emergency situations to reduce flood stage (*32*). Channel structures such as dikes, revetments, and levees also offer protection against high flood levels, although at the cost of reducing channel capacity and increasing river stage (*6*).

The 1955 design flood study accounted for flood regulation effects by incorporating 151 existing and planned reservoirs, selected on the basis of their storage capacity and potential impact on the Lower Mississippi River. The study simulated unregulated river flow in LMRB for design storm events, using unit hydrographs and the Puls routing method (*17*, *33*). For each reservoir, it estimated the maximum reduction in peak flow from the unregulated hydrograph, assuming full utilization of flood storage capacity. This approach resulted in a 9 to 13% reduction in peak discharge along the Lower Mississippi River (table S3). A more recent work, however, has argued that the 1955 study likely overestimated regulation effects due to its reliance on idealized storage utilization across all built and planned reservoirs (*17*).

A 2016 study conducted by the US Army Corps of Engineers (USACE) reassessed the 1955 design flood using modern hydrologic and hydraulic models. This includes implementing the National Weather Service (NWS) Community Hydrologic Prediction System framework that simulates SM, soil moisture, runoff generation, flood routing, and reservoir regulations over the MRB (*17*). This study accounted for regulation effects by modeling a smaller set of 94 reservoirs that were constructed by 2014, excluding unbuilt reservoirs from the 1955 study. Reservoir routing was performed using current water management plans, in which USACE hydrologists performed regulation based on simulated inflow and established rule curves, including joint operations for reservoirs in sequence. Regulated outflows were then fed back into the NWS model for final simulations. The 2016 study revealed a 3 to 5% reduction in peak discharge from flood regulation during the design storm event, along with higher peak magnitudes (e.g., 91,888 m^3^/s at Vicksburg compared to 83,818 m^3^/s in the 1955 study; see table S3).

Reservoir and floodway operational data over the MRB are managed by the USACE and unavailable to the public. There are also challenges implementing rule curves for individual reservoirs, which may require institutional collaboration across the MRB. Because of these constraints, this study simulated flood flows under unregulated conditions, assuming no influence from reservoirs or flood control structures. Notably, both the 1955 and 2016 studies suggest that regulation has limited impact on peak discharge during very extreme floods, such as the design flood. This low sensitivity to regulation indicates that the findings of increased flood hazard should still hold under regulated conditions.

Climate change impacts on extreme storm clusters pose challenges to the existing flood management system. The spatial pattern of major flood storage reservoirs in the MRB substantially influences flood control during extreme storm events (*17*, *34*). The upstream regions of Ohio-Tennessee and Arkansas River Basins contain more reservoirs and flood storage capacities than downstream areas (*31*, *35*, *36*). During a cluster of extreme storm events, not all upstream reservoir storage can be efficiently used, depending on the precipitation patterns and reservoir operating strategies. Because of the uncertainty in forecasting future storm events, it is challenging to perform optimized operation across the reservoir system to regulate extreme floods. Climate change can alter extreme storm cluster characteristics (Fig. 2) and precipitation spatiotemporal distributions (e.g., fig. S10), which further affects the effective use of reservoir storage. In addition, existing operational rules often lack consideration of these climate change impacts and need to be revised (*37*, *38*). It is worth conducting deeper research for existing reservoirs to account for storm clustering under future flood conditions, especially when viewed as a system rather than as independent units.

### Uncertainty analysis

One source of uncertainty in this study stems from the selection of GCMs. Our analyses were based on 38 ensembles from four GCMs under the SSP3-7.0 scenario. Using large ensembles is intended to better capture natural variability, generating more extreme flood scenarios for flood frequency and characteristics analysis compared to single-model simulations or small ensembles (*39*). These four GCMs are the only available CMIP6 models that provide 6-hour, 1° precipitation for SSP3-7.0 with more than five ensemble members. We highlight that high-resolution (6-hourly as compared to daily) and low-precipitation bias are critical factors for accurate flood simulations in the MRB, particularly for extreme events. The selected GCMs outperform others in these aspects (*40*–*42*), making them more suitable than models with coarser resolutions or higher biases. Our multimodel results reveal increasing trends in winter-spring extreme precipitation and peak discharge over the MRB, which is consistent with prior studies using multiple CMIP6 models (*10*, *41*, *43*, *44*). We suggest that using large ensembles from a few high-quality GCMs with lower biases can yield better flood simulations compared to a broader set of GCMs with coarser resolution and higher biases. However, incorporating multiple GCMs reduces model-specific biases and enhances robustness in trend estimation, highlighting a trade-off between GCM quantity and quality.

Another source of uncertainty relates to the GCMs’ representation of extreme rainfall. Historical observations have shown that flood-producing storms in the MRB are characterized by large areas of extreme convective rainfall organized along frontal boundaries, associated with water vapor flux from the Gulf of Mexico (*4*, *5*). The coarse resolution of the GCM data makes it difficult to explicitly represent fine-scale convective precipitation processes. This issue is addressed by applying a stochastic rainfall generator (see Materials and Methods) that simulates fine-scale precipitation fields conditioned on the large-scale GCM precipitation. The stochastic simulation accounts for the statistical relationship between fine-scale precipitation and GCM precipitation, as well as the space-time correlation structure of the convective precipitation systems. By conditioning on GCM data, our stochastic simulations can capture spatiotemporal heterogeneity in regional precipitation and climate change impacts within the study area. This idea of generating high-resolution weather predictions from coarse-scale models has been widely applied by recent studies for short-term weather forecasting and GCM downscaling, using deep learning, stochastic, or dynamic downscaling methods (*44*–*47*). Evaluation of annual maximum precipitation and storm sequence characteristics against observations demonstrate the rainfall generator’s ability to reproduce extreme precipitation characteristics over the MRB (see Supplementary Text). This evidence justifies the use of GCM precipitation as a basis for simulating extreme rainfall. Nevertheless, it should be noted that certain biases still exist in the GCM data (e.g., biases in storm space-time structures), which can introduce uncertainty in simulated storm events and the resulting flood scenarios.

The SSP3-7.0 scenario is a high greenhouse gas emission scenario characterized by a high level of radiative forcing and global warming (*48*). This scenario exhibits higher precipitation and temperature increases than the low (SSP1-2.6) or medium (SSP2-4.5) scenarios, despite being lower than the upper-end SSP5-8.5 scenario, which has been considered unlikely due to recent mitigation efforts (*49*). Therefore, the SSP3-7.0 scenario should be considered as the high end of plausible scenarios when assessing future flood hazard. It is anticipated that there will be smaller increases in extreme precipitation and flood magnitude in the LMRB under low or medium emission scenarios (*10*, *50*). Our study was limited to SSP3-7.0, however, due to the lack of GCM ensembles for other scenarios. The results from our study should therefore be interpreted as the upper end of plausible future flood changes; actual future flood likelihood may be lower with more aggressive climate change mitigation efforts. Future studies should aim to incorporate multiple emission scenarios to provide a more comprehensive assessment of future flood hazard in the LMRB (*51*). This will hinge upon the availability of additional large ensemble simulation results from CESM2 or other global models.

The hydrologic model introduces additional uncertainty. Although the HLM effectively reproduces peak discharge distributions, it tends to simulate slower receding limbs in flood hydrographs (see Supplementary Text and fig. S15). This is attributed to the model’s limitations in representing subsurface and groundwater processes. The HLM focuses on simulating precipitation-runoff and flood routing processes, which does not reflect comprehensive land surface-atmosphere interactions. Although extreme storm precipitation is the primary driver of major flood events, soil moisture-precipitation feedbacks can affect long-term basin water balance and can modulate the intensity of subsequent storms (*52*). The HLM also omits historical and future changes in river channels, land use, and flood regulation. Historical land use data show that, from 1949 to 2007, cropland decreased from 34 to 30% over the MRB, grassland and forest each remained at ~29%, and urban areas grew from 1 to 3% (table S4). This suggests minimal land use changes at the basin scale, but local shifts (e.g., urban expansion in floodplains) may influence flood generating process. For instance, there have been changes in agricultural practices, most notably the widespread introduction of subsurface agricultural drainage (i.e., tile drains) in some upper parts of the MRB (*53*). Although this study focuses on extreme storm impacts on flood hazards under unregulated conditions, we recognize that enhanced land use and flood mitigation strategies will play a critical role in reducing peak discharge and flood risks under future climate conditions and deserves future research. In addition, uncertainties from the observational datasets, the stochastic rainfall generator, and the GEV model also influence the results of this study.

## DISCUSSION

This study offers several key insights into extreme flood hazards in the LMRB. First, we demonstrated that major floods in the LMRB are driven by clusters of extreme storms rather than I events. Under future climate scenarios (2070 to 2100), we identified a shift in storm cluster characteristics preceding flood peaks, including a 26% increase in the number of storms, an 8% rise in average storm precipitation, and a 17% decrease in average dry intervals compared to 1990 to 2020. Storm clusters also exhibit enhanced future SC and TC, characterized by a growing number of affected sub-basins per storm and more recurring heavy precipitation over the same sub-basin.

Second, we classified four major flood types on the basis of storm clustering patterns: SC, TC, I, and CC. The CC type combines spatial and temporal storm clustering, characterized by recurring extreme storms delivering heavy precipitation across multiple sub-basins. This type generates the most severe flood conditions and exhibits the largest increase in peak discharge, 30-day peak volume, and flood duration under future climate scenarios. The dominant flood type is projected to shift from the I type (38% of events in 1990 to 2020) to the CC type (49% of events in 2070 to 2100), suggesting more frequent CC-type flooding with greater flood magnitude.

Third, we found that storm cluster precipitation is the primary contributor (70%) of increased peak discharge under future climate conditions, whereas wetter antecedent conditions also play an important role (57%). In contrast, a reduction in 30-day SM before flood peaks leads to a negative contribution to peak discharge change, although the influence is smaller (−27%).

Last, we projected that historically catastrophic floods will become more frequent under future climate conditions. The April 2011 flood is expected to shift from a 112-year event to a 23-year flood by 2050 and an 8-year flood by 2100. Similarly, the 1955 design flood (under unregulated conditions) is projected to have a return period of just 150 years by 2100, underscoring the inadequacy of current flood control measures to address future flood risks.

The critical role of storm clusters in driving major floods in the LMRB has profound implications for flood hazard prediction and management. Conventional precipitation and flood frequency analysis methods, which typically focus on single storm events [e.g., rainfall intensity-duration-frequency analysis; (*53*)], is inadequate for assessing flood hazards in large drainage basins like the LMRB. Instead, advanced approaches—such as rainfall-runoff models and stochastic methods, as used in this study—are necessary to capture the arrival and spatiotemporal patterns of storm clusters and to simulate flooding scenarios in large basins (*54*, *55*). Flood control strategies, including dam and spillway operations, must extend beyond mitigating the impacts of individual storm events to account for subsequent storms and the compounding effects of flood waves from tributaries. This underscores the need for long-range, accurate weather and flood forecasts. In addition, forecast-informed operational frameworks should be developed to incorporate the impacts of storm clustering to enhance flood management in the region.

## MATERIALS AND METHODS

### Datasets

The Analysis of Record for Calibration (AORC) dataset was used for precipitation observations, which provided 6-hour, 0.03° total precipitation depth from 1979 to 2021. The AORC was created on the basis of NLDAS-2 and Stage IV precipitation datasets (*56*). We also used 6-hour, 0.25° large-scale precipitation, 2-m temperature, and monthly evapotranspiration for the same time period from the ERA5 reanalysis dataset (*57*). Time series of daily discharge observations and annual peaks at four US Geological Survey (USGS) gauges in the Lower Mississippi River were obtained from the USGS National Water Information System (*58*), including Vicksburg (USGS 07289000), Arkansas City (USGS 07265450), Helena (USGS 07047970), and Memphis (USGS 07032000).

We used multiensemble simulations from four GCMs in the Coupled Model Intercomparison Project (CMIP6): Community Earth System Model 2 [CESM2; 10 ensemble members; (*39*)], Energy Exascale Earth System Model [E3SM; 10 ensemble members; (*42*)]; Max Planck Institute for Meteorology Earth System Model [MPI-ESM1-2-HR; 10 ensemble members; (*59*)]; and EC-Earth3 [8 ensemble members; (*60*)]. This yields 38 ensemble members, each providing 6-hour, 1° large-scale precipitation, daily 2-m temperature, and monthly evapotranspiration. The GCM data span historical (1901 to 2014) and future periods (2015 to 2100) under the SSP3-7.0 scenario.

The GCM precipitation and evapotranspiration were bias corrected against ERA5 reanalysis data (upscaled to match the GCM resolution) for each season using the CDF-t method (*61*). The CDF-t method uses linear interpolation to obtain a transformation *T* that maps the cumulative distribution function (CDF) of a GCM variable (*F_GCM,h_*) to the corresponding CDF from ERA5 (*F_ERA5,h_*) in the historical period (1979 to 2021). The transformation *T* is then applied to the GCM CDF in the early (1901 to 1978) or future (2022 to 2100) periods, denoted by *F_GCM,f_*, to generate an adjusted CDF *F_ERA5,f_* that represents the “unobserved” ERA5 CDF for those periods (*28*). For the historical period, quantile-quantile (Q-Q) mapping between *F_GCM,h_* and *F_ERA5,h_* is used to correct the GCM data. For the early and future periods, Q-Q mapping is applied between *F_GCM,f_* and *F_ERA5,f_*. The CDF-t method accounts for CDF changes between historical and future GCM simulations, thus preserving the trend in GCM data while correcting bias.

### Storm tracking

A storm tracking algorithm called STARCH (*30*) was used to identify storm events in the MRB. The algorithm identified continuous regions of precipitation greater than 1 mm/hour as individual storm objects at each time step. The identified storm objects at consecutive time steps were tracked as a single event if their overlapping ratio is greater than 0.2

$$R\left(t_{2},i,j\right)=\frac{A_{0}}{A_{i}\left(t_{2}\right)}+\frac{A_{0}}{A_{j}\left(t_{1}\right)}$$ (1)

where $R\left(t_{2},i,j\right)\subset \left[0,2\right]$ is the overlapping ratio, *A_j_*(*t*_1_) is the area of storm object *j* at time step *t_1_*, *A_i_*(*t*_2_) is the area of storm object *i* at time step *t*_2_, and *A*_0_ is the overlapping area between storm objects *i* and *j*. The storm tracking was applied to GCM precipitation data, showing good accuracy in identifying the start and end of storm events in the basin (see Supplementary Text).

### Stochastic precipitation simulation

We used a stochastic rainfall generator called StormLab to generate high-resolution precipitation fields (6-hour, 0.03° resolution) conditioned on coarse-scale GCM precipitation data over the MRB (*28*). The process involved three major steps:

1) Time-varying distribution fitting:

Precipitation distributions were fitted at each 0.03° grid cell in the study domain (29°N to 50°N, 79°W to 113°W, consisting of 1024 × 630 grids). The distribution consists of two components:

(i) The probability of precipitation occurrence $P_{\mathrm{wet}}\left(t\right)$ was modeled using logistic regression (*62*)

$$P_{\mathrm{wet}}\left(t\right)=\frac{1}{1+\text{exp}\left\{-\left[\alpha_{0}+\alpha_{1}PR\left(t\right)\right]\right\}}$$ (2)

where PR(t) is large-scale precipitation linearly interpolated to the 0.03° grid, and $\alpha_{0}$ and $\alpha_{1}$ are fitted parameters.

(ii) The precipitation amount, i.e., the “nonzero” part of the precipitation series, was modeled by a nonstationary generalized gamma distribution (*28*). The precipitation amount *x* is assumed to follow a generalized gamma distribution with shape parameter *c* > 0 (*63*). We then modeled the mean and variance of the transformed variable $y=x^{c}$ as functions of the large-scale precipitation

$$\begin{array}{c} \mu \left(t\right)=\frac{\mu_{c}}{\beta_{0}}\text{log}\left\{1+\left[\text{exp}\left(\beta_{0}\right)-1\right]\left[\beta_{1}+\beta_{2}\mathrm{PR}\left(t\right)\right]\right\} \end{array}$$ (3)

$$\sigma^{2}\left(t\right)=\beta_{4}\sigma_{c}^{2}\left[\frac{\mu \left(t\right)}{\mu_{c}}\right]$$ (4)

where $\mu_{c}$ and $\sigma_{c}^{2}$ are the mean and variance of a stationary generalized gamma distribution fitted to historical AORC precipitation data at the grid, and $\beta_{0},\beta_{1},\beta_{2},\beta_{3}$ and $\beta_{4}$ are fitted parameters. The parameterization in Eq. 3 covers a linear to a logarithmic relationship between the mean μ(t) and large-scale precipitation PR(t). Equation 4 assumes the variance $\sigma^{2}\left(t\right)$ changes linearly with the mean, moderated by the parameter $\beta_{4}$.

The precipitation distributions were fitted by season on the basis of AORC and ERA5 large-scale precipitation from 1979–2021 at each grid cell in the study domain. The Python Scikit-learn package was used to estimate the logistic regression parameters using maximum likelihood (*64*). The precipitation amount distribution was fitted by minimizing the mean continuous ranked probability score [CRPS, (*65*)] using the optimization function in Scipy (*66*).

2) Noise generation: At each GCM time step, we extracted a high-resolution precipitation field from historical AORC data that best matched the current GCM precipitation field. This match was determined by minimizing the L2 distance between the GCM and the AORC precipitation fields (upscaled to GCM spatial resolution). To account for seasonality, only AORC precipitation fields from the same or adjacent months to the current GCM time step were considered in the matching process. Next, we divided the matched AORC precipitation field into overlapping local windows of size (128, 128) grid cells, with a 30% overlap between neighboring windows. For each window, we calculated the amplitude spectra of the AORC precipitation and simulated spatially correlated Gaussian noise fields using an inverse fast Fourier transform (*67*). The simulated noise fields exhibit the local spatial structure of the AORC precipitation within each window. The final noise was obtained by aggregating the local noise from all the windows. The temporal evolution of the noise field was modeled as a lag-1 autoregressive process

$$W\left(t+1\right)=\rho W\left(t\right)+\sqrt{1-\rho^{2}}\tilde{W}\left(t+1\right)$$ (5)

where W(t+1) is the noise field at time *t* + 1, W(t) is the noise field at time *t*, and $\tilde{W}\left(t+1\right)$ is the new spatially correlated noise field generated from the matched AORC precipitation field at time *t* + 1. ρ is the correlation coefficient between GCM precipitation at time *t* and *t* + 1.

The generated noise fields incorporate the amplitude spectrum of the matched AORC precipitation fields while discarding the phase information, i.e., exact precipitation locations and depths. As a result, these noise fields can be considered as random realizations of the possible patterns of local-scale precipitation. This method aims to identify the potential spatiotemporal structure of local-scale precipitation corresponding to the GCM coarse-scale precipitation fields, based on the assumption that storms with similar large-scale precipitation patterns exhibit similar local-scale spatial correlation structures. Generating noise fields based on local windows was intended to capture the spatial variability of precipitation patterns, which are particularly important in large river basins.

3) Noise-to-precipitation conversion: This step involves converting the simulated noise fields into precipitation fields. The GCM coarse-scale precipitation fields were interpolated to 0.03° grids and used as PR(t) in Eqs. 3 and 4 to calculate the spatial fields of $P_{\mathrm{wet}}\left(t\right)$ and parameters of the precipitation amount distribution. Next, the noise fields were converted to probability fields by finding the corresponding probabilities of the noise values from the standard Gaussian CDF. For each grid cell, if the converted probability P(t) was lower than the dry probability $P_{\mathrm{dry}}\left(t\right)=1-P_{\mathrm{wet}}\left(t\right)$ , the simulated precipitation was set to zero. If $P\left(t\right)\geq P_{\mathrm{dry}}\left(t\right)$ , the excess probability was rescaled to the range [0, 1] as $P^{\prime }\left(t\right)=\frac{P\left(t\right)-P_{\mathrm{dry}}\left(t\right)}{1-P_{\mathrm{dry}}\left(t\right)}$ . The rescaled probability $P^{\prime }\left(t\right)$ was used to obtain the simulated precipitation values from the inverse of the precipitation amount CDF.

StormLab was used to simulate high-resolution precipitation fields conditioned on the 38 GCM ensemble members for the 1901 to 2100 period. The simulation was performed on an event basis. Storms were first identified from the GCM data using the storm tracking algorithm. StormLab was then applied to simulate high-resolution precipitation fields for each identified storm event. It should be noted that StormLab does not distinguish the form of precipitation (i.e., rain or snow), which was taken into account in the hydrological modeling (described in the next section). One major source of uncertainty in StormLab simulation arises from the transition from ERA5 to GCM—whereas the precipitation distributions were fitted on the basis of AORC data and ERA5 large-scale precipitation, the GCM data were used as covariates in precipitation simulation. This uncertainty is reduced by bias correcting GCM precipitation against ERA5 (described in Materials and Methods). Discrepancies may remain between the two datasets; however, such as biases in space-time correlation structures of the precipitation patterns. Notwithstanding possible discrepancies, we evaluated the StormLab simulations against the AORC data and found good consistency in annual maximum precipitation and precipitation climatology in the MRB (see Supplementary Text), which justifies the use of bias-corrected GCM data in simulating precipitation.

### Hydrologic modeling

The HLM was used to simulate the discharge response in the LMRB (*68*). The HLM has been successfully implemented for flood forecasting in watersheds within the MRB, demonstrating high skill in simulating streamflow fluctuations and annual maxima (*69*–*73*). The model represents a river basin by decomposing it into hillslopes and river channels. The hillslope serves as the basic hydrologic response unit for the HLM, which simulates hydrological processes including SM, runoff generation, evapotranspiration, infiltration, and percolation (*68*). The simulated streamflow from each hillslope is then routed through the basin river network. The model can efficiently simulate complex hydrological processes and discharge responses in large drainage basins like the MRB (*73*).

The HLM simulation domain was set as the entire MRB, with a river network obtained from the HydroRIVERS dataset (*74*). The MRB, which spans an area of 2,980,000 km^2^, was divided into 170,530 hillslopes connected by river channels, with an average area of 18.6 km^2^. The model requires three forcings: (i) 6-hour gridded precipitation depth, (ii) daily gridded surface temperature, and (iii) monthly gridded evapotranspiration. Each hillslope unit was assigned precipitation depth, temperature, and evapotranspiration from the closest grid cell to the hillslope center. The precipitation was considered as snowfall if the surface temperature fell below the threshold of 0°C. The SM process begins when the surface temperature rises above 0°C, i.e., the degree-day method (*75*). The model outputs are daily discharge time series at key gauge locations along the Lower Mississippi River.

Previous MRB design flood studies modeled flood flows under both “unregulated” and “regulated” conditions (*17*). The “unregulated” condition assumed no influence from upstream reservoirs and downstream flood control structures, whereas the “regulated” condition considered existing and near future reservoirs and floodway operations. Because of the lack of access to reservoir and flood control structure operational data, this study simulated “unregulated” flood conditions. We focused on assessing peak discharge at four gauges along the Lower Mississippi River: Memphis, Helena, Arkansas City, and Vicksburg (fig. S1). Gauges located downstream of the Old River Control Structure were not included in the analyses.

The HLM was calibrated by tuning the model parameters on the basis of the parameter settings from previous modeling efforts over the MRB (*70*, *73*), ensuring the model simulations fit the available daily discharge observations at Vicksburg (2008 to 2021) and Memphis (2014 to 2021). The historical discharge was simulated using AORC precipitation (6-hour, 0.03° resolution), ERA5 surface temperature (daily, 0.25°), and ERA5 evapotranspiration (monthly, 0.25°). The simulations were performed for each year individually via parallel computing to save computational time. A spin-up year was added before each simulation, using forcings of the previous year and zero initial conditions. The simulated discharge in the spin-up year was discarded in each run. For simplicity, we used the same set of model parameters in each major sub-basin in the MRB (table S2). The calibrated model’s simulations agree well with discharge observations at the two gauges in the Lower Mississippi River (fig. S13), with Nash-Sutcliffe efficiency (NSE) values of 0.69 and 0.66 at Vicksburg and Memphis, respectively. Although assuming uniform model parameters can result in biases local discharge response (e.g., in upstream areas of the MRB), the good agreement with observations suggests that the calibrated model is sufficient to represent flood discharge in the Lower Mississippi River.

The calibrated HLM was used to simulate discharge response on the basis of StormLab-simulated 6-hour, 0.03° precipitation generated from 38 GCM ensembles from 1901 to 2100. Other forcings include surface temperature (daily, 1°) and evapotranspiration (monthly, 1°) from GCM data. The simulation was performed individually for each year with an additional spin-up year, similar to the calibration run. We used the average basin condition of historical runs from 1979 to 2021 on 1 January as the initial condition for the spin-up year. The outputs were discharge time series at the four gauges from 1901 to 2100 across the 38 GCM ensembles. We focused our analysis on the annual peak events occurring in winter and spring seasons (December to May) for each year, which are major flood seasons and account for >90% of the historical annual peak events (table S1). The empirical return level plots (fig. S14) and Kolmogorov-Smirnov (K-S) tests show that the simulated peak discharge aligns closely with gauge observations, indicating that our approach can reproduce the observed distributions of winter-spring peaks in the LMRB (see Supplementary Text).

### Extreme storm cluster analysis

We examined the characteristics of extreme storm clusters preceding winter-spring peak discharge at Vicksburg. We focused on storm events within 30 days before the flood peak. The 30-day window was selected to capture the cumulative precipitation and storm interactions that contribute to LMRB floods as historical floods are typically driven by storm events occurred within 30 days before peak discharge (*4*, *17*). For each storm event, we calculated total precipitation across the five major sub-basins. A storm was classified as producing heavy precipitation in a sub-basin if its precipitation exceeded the 90th percentile of historical storm records (1979 to 2021) for that sub-basin. Extreme storms were defined as events causing heavy precipitation in at least one sub-basin. This criterion is designed to account for regional rainfall variability and to highlight storm’s localized impacts across sub-basins. Other storm events that did not meet this criterion were referred to as nonextreme storms.

We quantified key storm cluster characteristics within the 30-day period: the number of extreme storms, average storm precipitation, total accumulated precipitation, average duration, average storm area, and average interval between extreme storms. We introduced the storm SC metric *N_s_*, defined as the maximum number of sub-basins experiencing co-occurring heavy precipitation from a single storm event within the 30-day window. For TC, we defined the metric *N_t_* as the number of recurring storms within the 30-day period. Storm events are considered recurring if they produce heavy precipitation in the same sub-basin within a 7-day interval. The choice of 7-day focuses on storm compounding at the scale of days to weeks, where successive storms can exacerbate impacts on flooding and soil saturation before the affected sub-basin fully recovers. These characteristics were compared between historical (1990 to 2020) and future (2070 to 2100) periods. A sensitivity analysis was performed by varying the heavy precipitation threshold from the 85th to 95th percentile. This revealed moderate changes in the magnitude of storm cluster characteristics but did not affect the overall trends.

### Flood event analysis

We calculated winter-spring flood peaks and 30-day peak volumes at Vicksburg for historical (1990 to 2020) and future (2070 to 2100) periods using discharge simulations. Flood event duration was defined as the time that flood hydrograph exceeded the “major flood level” at Vicksburg—50 feet or 54,000 m^3^/s based on the rating curve (*58*). The major flood level was obtained from the US National Water Prediction Services (*76*). This threshold indicates extensive infrastructure inundation and massive resident evacuations.

Flood events were classified into four types on the basis of extreme storm clustering patterns within the 30 days preceding the peak: SC, TC, I, and CC. These types are defined as follows:

1) SC: Represents a single extreme storm causing heavy precipitation across multiple sub-basins. Classification requires the SC metric (*N_s_*) ≥ 3—indicating that a storm event produced heavy precipitation in at least three sub-basins. Also, the TC metric (*N_t_*) < 2, showing that no recurring extreme storms in the same sub-basin.

2) TC: Represents recurring extreme storms producing heavy precipitation in one or two sub-basins. Requires *N_s_* < 3 (weak or moderate SC) and *N_t_* ≥ 2 (recurring extreme storms in the same sub-basin).

3) I: Indicates one storm producing heavy precipitation in one or two sub-basins, with weak or moderate precipitation elsewhere. Requires *N_s_* < 3 and *N_t_* < 2, reflecting minimal SC and TC.

4) CC: Combines SC and TC, where multiple extreme storms occur in rapid succession, each affecting multiple sub-basins. Requires *N_s_* ≥ 3 and *N_t_* ≥ 2, indicating widespread and recurring heavy precipitation.

We calculated the relative proportion of each flood type in the historical and future periods and analyzed changes in key flood characteristics (e.g., peak discharge, volume, and duration) across these types.

We investigated the contributions of storm precipitation, basin antecedent conditions, and SM to changes in peak discharge. We defined the SAF as the percentage of basin areas that have static tank storage ≥ 90% of maximum capacity on the 30th day before the peak (smoothed with a 7-day moving average). In the HLM, the static tank storage reflects surface soil moisture, with high values indicating saturated soils. The SAF thus represents the basin’s antecedent wetness before the arrival of extreme storm clusters. We used a linear model to quantify the relationships between peak discharge and the three contributing factors

$$Q=\beta_{0}+\beta_{1}P_{30}+\beta_{2}\text{SAF}+\beta_{3}\text{SM}+\varepsilon$$ (6)

where *Q* is the winter-spring peak discharge, P_30_ and SM are the total precipitation and snowmelt within the 30 days before the peak, SAF is the saturated area fraction, β_0_, …, β_3_ are regression coefficients, and ε is the error term. Total P_30_ was calculated from StormLab simulations, and accumulated SM was calculated from HLM’s snow storage outputs.

The linear model was fitted to simulate flood events from the historical period (1990 to 2020). Parameter uncertainty was assessed via bootstrapping, refitting the model 1000 times with resampled flood event data (with replacement). We then calculated ∆P_30_, ∆SAF, and ∆SM, representing the differences in averages of these factors between historical (1990 to 2020) and future (2070 to 2100) periods. The contribution of precipitation to changes in peak discharge (∆*Q*) was estimated as $\frac{\beta_{1}\Delta P_{30}}{\Delta Q}\times 100\%$ , where ∆*Q* is the change in mean peak discharge predicted by the linear model

$$\Delta Q=\beta_{0}+\beta_{1}\Delta P_{30}+\beta_{2}\Delta \text{SAF}+\beta_{3}\Delta \text{SM}$$ (7)

Contributions of SAF and SM were calculated in a similar manner. Note that this model captures only linear contributions from the three factors. Nonlinear relationships, including interactions between factors, require more complex models.

### Extreme value analysis

The simulated winter-spring annual peak discharge was fitted via a generalized additive model of the GEV distribution, with the location and scale parameters modeled as smooth functions of year using cubic regression splines (*77*)

$$\begin{array}{c} y∼\text{GEV}\left[\mu \left(t\right),\sigma \left(t\right),\xi \right]\\ \mu \left(t\right)=\beta_{0}+\sum_{j=1}^{k}\beta_{j}b_{j}\left(t\right)\\ \sigma \left(t\right)=\text{exp}\left[\gamma_{0}+\sum_{j=1}^{k}\gamma_{j}c_{j}\left(t\right)\right] \end{array}$$ (8)

where *y* is the annual peak discharge, *t* is the predictor variable (i.e., year), μ(t) is the location parameter, σ(t) is the scale parameter, and ξ is the shape parameter. The parameters β_0_ and γ_0_ are intercept terms, whereas β*_j_* and γ*_j_* are coefficients associated with the cubic regression spline basis functions *b_j_*(*t*) and *c_j_*(*t*). The parameter *k* is the number of knots, which is chosen as 10. The exponential function exp() was used to ensure that the scale parameter is always positive.

The cubic regression spline divides the range of years (1901 to 2100) into *k* even periods and fits a cubic polynomial to the samples within each period. The cubic polynomials are then connected to form a piecewise cubic function, which approximates the nonlinear relationship between the response variables [i.e., μ(t) and σ(t)] and year *t*. The GEV model was fitted to all simulated flood peaks from 1901 to 2100 at each gauge location by penalized maximum likelihood estimation using the R package “mgcv” (*78*). It should be noted that there were 38 flood peak realizations per model year from the GCM ensembles, all of which were used to fit a single nonstationary GEV model. This implementation meets the GEV block maxima assumption because the 38 flood peaks in each model year were drawn from distinct discharge simulations conditioned on different GCM ensemble members, which can be considered as individual block maxima. The intention is to fit the GEV model with a large sample size of flood events, which can greatly reduce the uncertainty in estimating very extreme events [e.g., the 1000-year flood; (*31*)]. We used bootstrapping to estimate parameter uncertainty. A bootstrap sample was created by sampling 38 peak discharge simulations with replacement for each year from 1901 to 2100, which was then used to fit a GEV model. The bootstrapping process was repeated 1000 times to obtain the range of model parameters.

The shape parameter ξ was assumed to be constant due to the high uncertainties and complexity of estimating a time-varying shape parameter [e.g., (*53*, *79*–*82*)]. This assumption simplifies the model but may underestimate future flood frequency and magnitude if the distribution shifts toward a heavier tail under future climate conditions. We also evaluated a stationary model and a model with only the location parameter as a function of year. The model with nonstationary location and scale had the lowest average Akaike Information Criterion (fig. S16), suggesting a better fit to the peak discharge data. The same model was also selected on the basis of Bayesian Information Criterion. A sensitivity analysis was performed by changing the number of knots from 6 to 14, which showed minor influence on model fitting. Evaluation based on Q-Q plots showed good fitting of the GEV model to simulated flood peaks (see Supplementary Text). On the basis of the fitted GEV models, we estimated the equivalent return periods of the 1927, 1937, and 2011 Mississippi Floods and the 1955 design flood for each year from 1901 to 2100. The return level curves were estimated for the years 2020, 2050, 2080, and 2100. The design flood peak discharge was obtained from the 1955 design flood study under the “unregulated” condition (e.g., 83,818 m^3^/s at Vicksburg). A recent reassessment of the design flood in 2016 estimated higher peak values, e.g., 91,888 m^3^/s for Vicksburg (*17*). However, we chose the peak values from the 1955 study because they were used as the design capacity of existing flood management infrastructure and decision systems.
