# Supplementary material for: Increasing flood hazard in the Lower Mississippi River due to extreme storm clustering
Source: Sci Adv. 2025 Oct 1;11(40):eadt1868. doi: 10.1126/sciadv.adt1868 (PMC12487879; doi:10.1126/sciadv.adt1868)
Supplement: Supplementary file 1 — Supplementary Text Figs. S1 to S17 Tables S1 to S4 [file sciadv.adt1868_sm.pdf]

Science Advances
AAAS

Supplementary Materials for

Increasing flood hazard in the Lower Mississippi River due to extreme storm clustering

Yuan Liu *et al.*

Corresponding author: Daniel B. Wright, danielb.wright@wisc.edu

Sci. Adv. **11**, eadt1868 (2025)
DOI: 10.1126/sciadv.adt1868

This PDF file includes:

Supplementary Text
Figs. S1 to S17
Tables S1 to S4

Supplementary Text

Validation of storm tracking and StormLab precipitation simulation

Fig. S8 illustrates an example of a simulated storm event over the Mississippi River Basin. The storm tracking algorithm successfully tracked the lifetime of the storm object and associated GCM precipitation patterns. The high-resolution precipitation fields generated from StormLab exhibited similar patterns to the original GCM precipitation but with more fine-scale variability. To assess the performance of StormLab simulations, we calculated annual maximum winter-spring precipitation (December-May) for durations from 1 to 15 days in the five major sub-basins, using the Analysis of Record for Calibration (AORC) data (1990-2020) and StormLab simulations for historical (1990-2020) and future (2070-2100) periods (Fig. S9). The simulated 3-, 7-, and 15-day maximum precipitation showed good agreement with AORC data in the Lower Mississippi River, Ohio-Tennessee River, and Upper Mississippi River Basins, although the 1-day precipitation was slightly overestimated. StormLab generated lower extreme precipitation in the Arkansas-Red and Missouri River Basins, possibly due to the remaining biases in the CESM2 data. Uncertainties in StormLab's parameterization and noise generation can also contribute to this discrepancy. This includes uncertainty in precipitation distribution fitting and biases in precipitation temporal autocorrelation structures, which are important factors for short-duration extremes. This underestimation had a minor impact on simulated peak discharge because these two drainage basins have a relatively smaller contribution to extreme floods in the Lower Mississippi River compared to the other three sub-basins. In the future period, an overall increase in winter-spring maximum precipitation was found across different durations, supporting the intensification of extreme storm clustering in the Mississippi River Basin.

We calculated the average winter-spring total precipitation in the Mississippi River Basin based on AORC data (1990-2020) and StormLab simulations for historical (1990-2020) and future (2070-2100) periods (Fig. S10). The StormLab simulations agreed well with the AORC data for the 1990-2020 period, with an average difference of 1% across grid cells in the Mississippi River Basin. The result shows an overall increase in winter-spring total precipitation under future climate conditions. Similar patterns of increased precipitation can be found from the four GCMs (Fig. S11), although the MPI-HR model exhibited relatively smaller increase and reduced precipitation over the Arkansas-Red River Basin specifically.

We also calculated the characteristics of extreme storm clusters preceding historical flood peaks using AORC data from 1990 to 2020. These characteristics were then compared with those derived from StormLab simulations for the same period (Fig. S12). Results show that StormLab's simulated storm characteristics generally align well with AORC data, except for a slight underestimation of average extreme storm precipitation. We attribute this discrepancy to potential remaining biases within the GCM data and the limitations of StormLab's parameterization. The parametric equations describing precipitation distribution (Eqs. 3-4) may not fully represent the complex relationship between local and large-scale precipitation. Additionally, the simplification of temporal noise evolution as a lag-1 autoregressive process might underestimate temporal autocorrelation of extreme precipitation, leading to lower simulated extreme values.

Validation of Hillslope Link Model simulation

To evaluate the performance of the simulation approach used in this study, we compared the empirical return level curves of simulated winter-spring peak discharge with available stream gauge observations from 1901 to 2020 (Fig. S14). Results show that simulated discharges align well with gauge observations at the four gauges in the Lower Mississippi River. We used Kolmogorov-Smirnov (K-S) test to compare cumulative distribution functions (CDFs) between the two series: Vicksburg (K-S statistic=0.09; p=0.50); Arkansas City (K-S statistic=0.06; p=0.93); Helena (K-S statistic=0.11; p=0.36); Memphis (K-S statistic=0.13; p=0.22). High p-values (e.g., above 0.05) indicate that the K-S test found no significant difference between the simulated and observed flood peak CDFs. These results suggest that the stochastic rainfall simulation and hydrologic model used in this study can reproduce the distributions of winter-spring peak discharge at these locations.

We also calculated the slopes of the rising and receding limbs of the observed and simulated flood hydrographs. These slopes are defined as the rate of discharge change from 30 days before or after the peak time, relative to the peak discharge. While the simulated hydrograph exhibits rising slopes comparable to observations, its receding slopes are relatively smaller (Fig. S15). This slower recession can also be seen in historical simulations (Fig. S13). We attribute this to the limitations of the HLM in representing subsurface and groundwater processes, which causes an earlier and quicker return of infiltrated and percolated water to river channels. Nevertheless, these differences have minimal impacts on the study's key findings, which focus on flood peak discharge.

Validation of GEV fitting

Quantile-quantile (Q-Q) plots were created to evaluate the fitting of the nonstationary GEV model. We used the fitted GEV model to simulate 38 peak discharge values (one for each ensemble member) for each year from 1901 to 2100. The simulated peak discharge series were sorted and plotted against the peak discharge data simulated by the Hillslope Link Model (HLM) at the corresponding quantiles. We repeated the GEV simulation 1,000 times and used the median peak discharge values at each quantile to plot against the HLM-simulated peak discharge (Fig. S17). The Q-Q plots demonstrate that the nonstationary GEV model provides a good fit to the HLM-simulated peak discharge at the four gauges considered in this study.

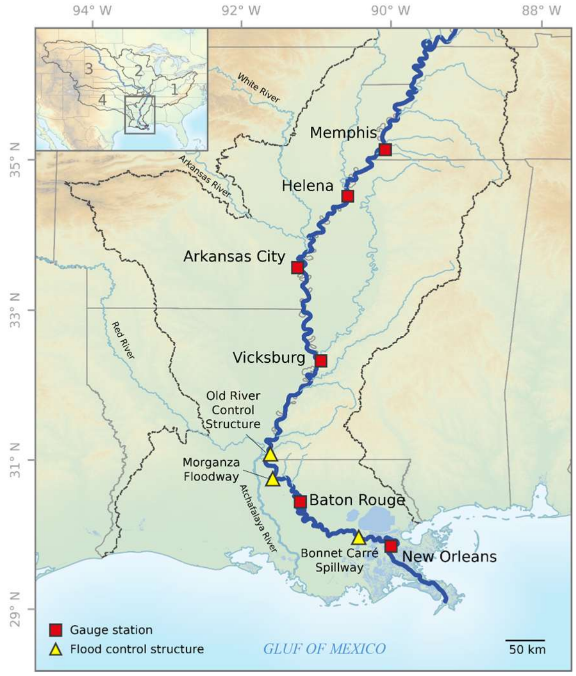

Fig. S1 The Lower Mississippi River Basin. The dark blue line denotes the Mississippi River. The background color represents relative land surface elevation. The map in the upper left corner shows the entire Mississippi River Basin and its five major sub-basins: (1) Ohio-Tennessee, (2) Upper Mississippi, (3) Missouri, (4) Arkansas-Red, and (box region) Lower Mississippi.

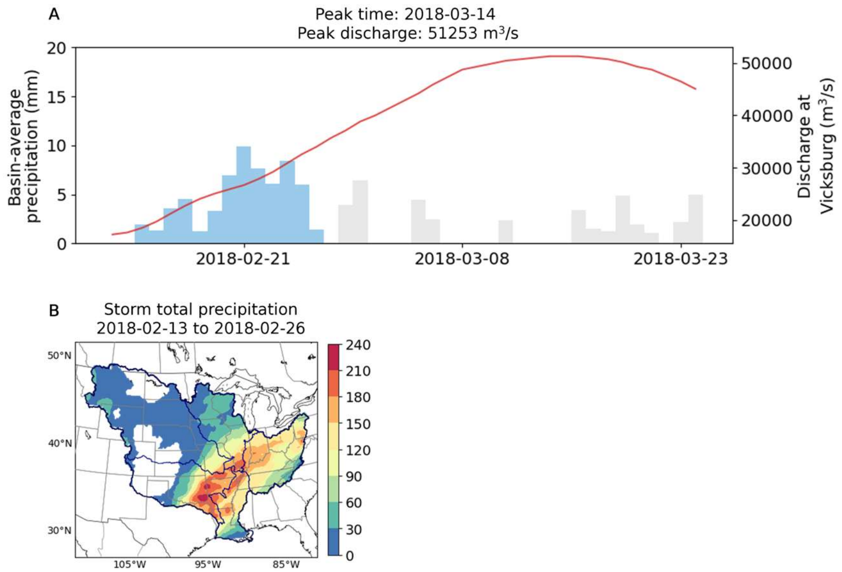

Fig. S2 Observed hydrograph of the February 2018 flood and extreme storm events preceding the flood peak. A, Flood hydrograph at Vicksburg (red line) and daily AORC precipitation (gray bar). Extreme storm events are highlighted in different colors. **B,** Total precipitation patterns of extreme storm events corresponding to each colored period in (**A**).

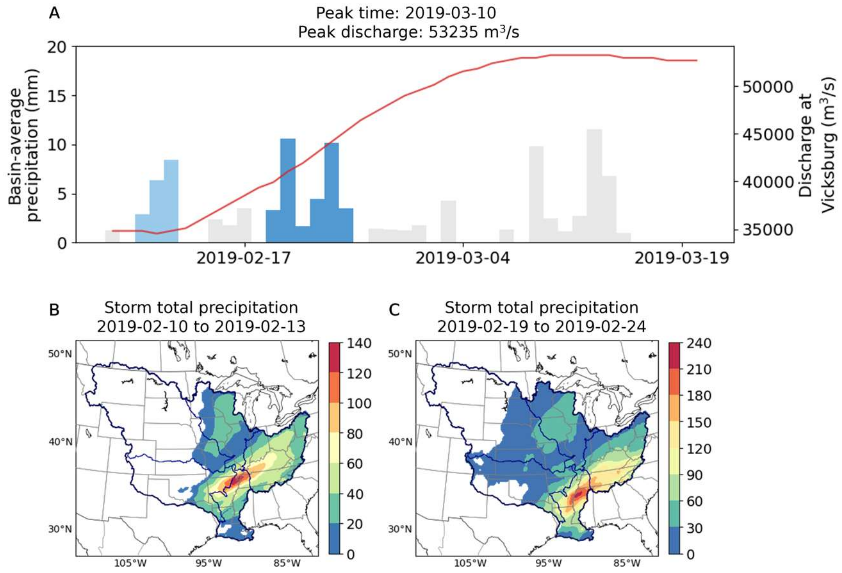

Fig. S3 Observed hydrograph of the March 2019 flood and extreme storm events preceding the flood peak. A, Flood hydrograph at Vicksburg (red line) and daily AORC precipitation (gray bar). Extreme storm events are highlighted in different colors. **B-C,** Total precipitation patterns of extreme storm events corresponding to each colored period in (**A**).

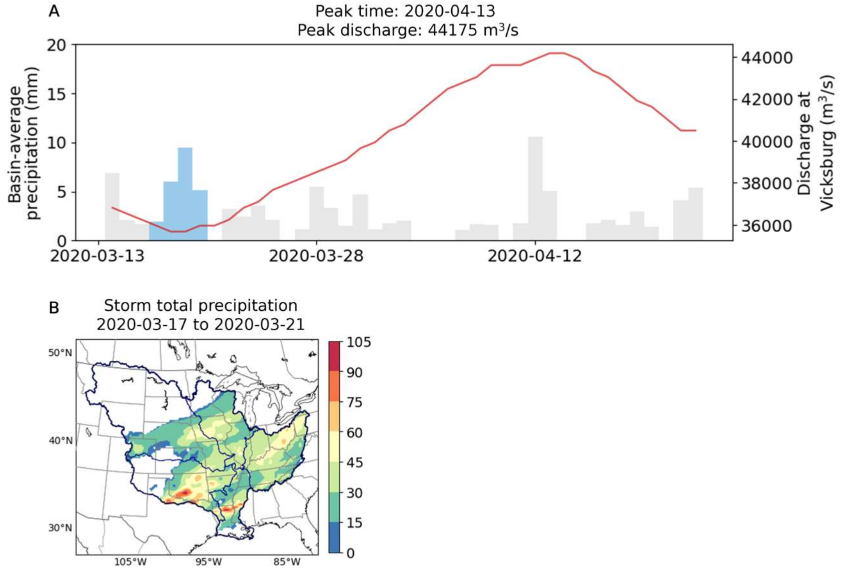

Fig. S4 Observed hydrograph of the March 2020 flood and extreme storm events preceding the flood peak. A, Flood hydrograph at Vicksburg (red line) and daily AORC precipitation (gray bar). Extreme storm events are highlighted in different colors. **B,** Total precipitation patterns of extreme storm events corresponding to each colored period in (**A**).

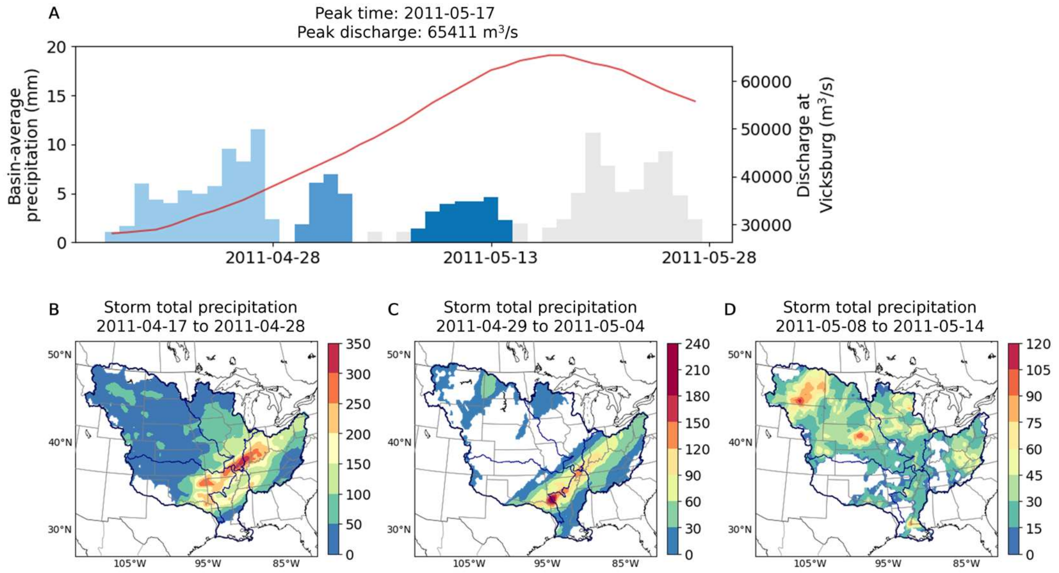

Fig. S5 Observed hydrograph of the April 2011 flood and extreme storm events preceding the flood peak. A, Flood hydrograph at Vicksburg (red line) and daily AORC precipitation (gray bar). Extreme storm events are highlighted in different colors. **B-D,** Total precipitation patterns of extreme storm events corresponding to each colored period in (**A**).

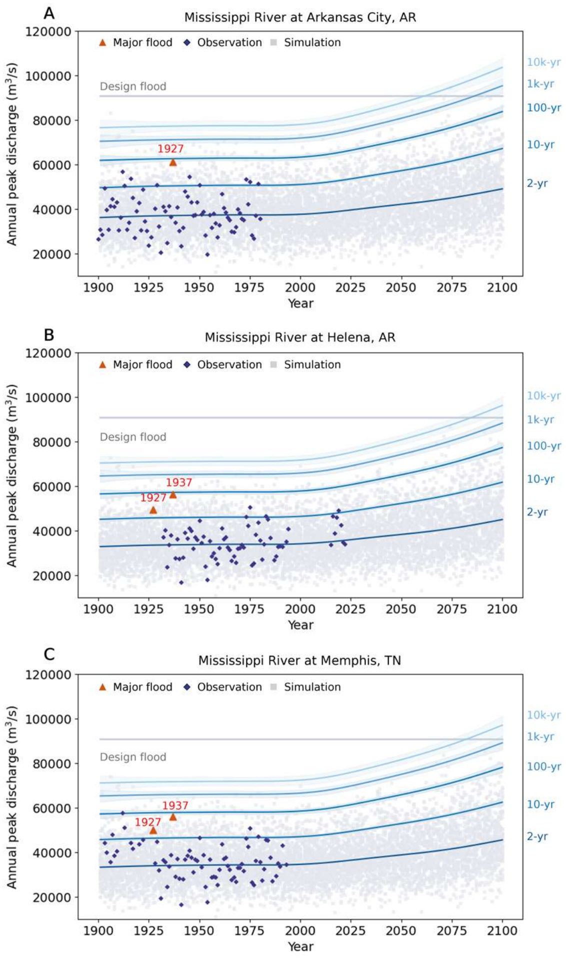

Fig S6. Changes in return levels of extreme winter-spring floods. A, Arkansas City, **B,** Helena, **C,** Memphis. Winter-spring peak discharge for return periods of 2 to 10,000 years (solid lines), with 95% prediction intervals (shaded areas). Purple diamonds represent peak discharge observations, with major flood events marked by red triangles. Gray dots represent simulated peak discharges from 1901 to 2100. The horizontal gray line denotes the 1955 design flood peak discharge.

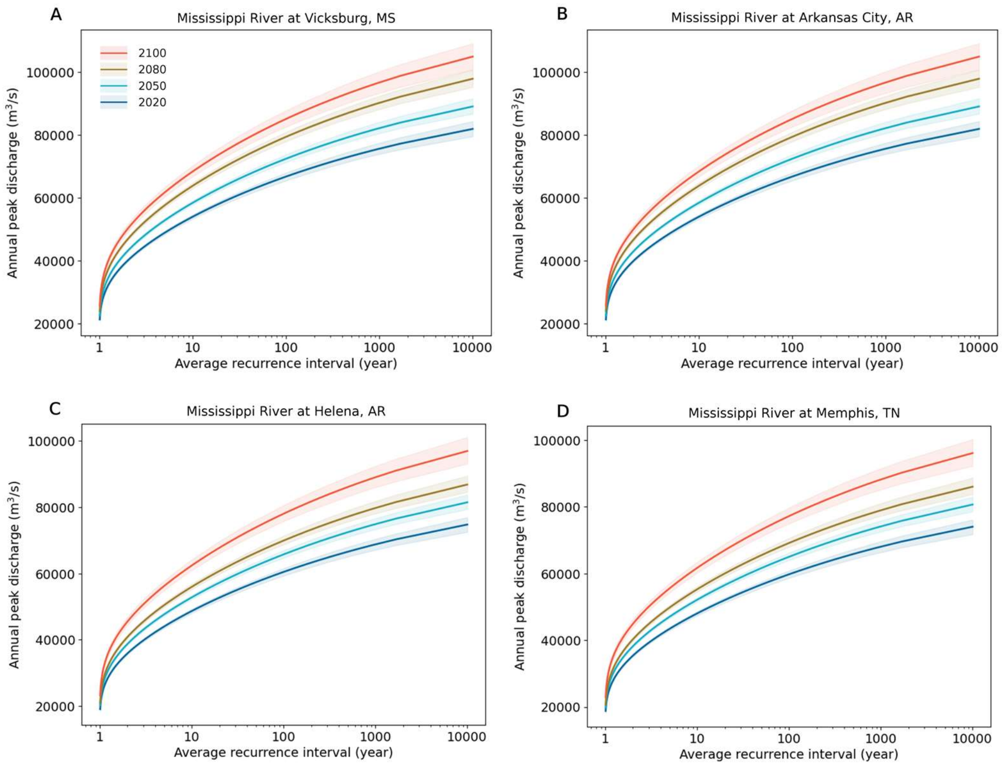

Fig. S7 Return level curves of winter-spring peak discharge for selected years: 2020 (blue), 2050 (aqua), 2080 (yellow), and 2100 (red). A, Vicksburg, **B,** Arkansas City, **C,** Helena, **D,** Memphis. Shaded areas represent 95% prediction intervals.

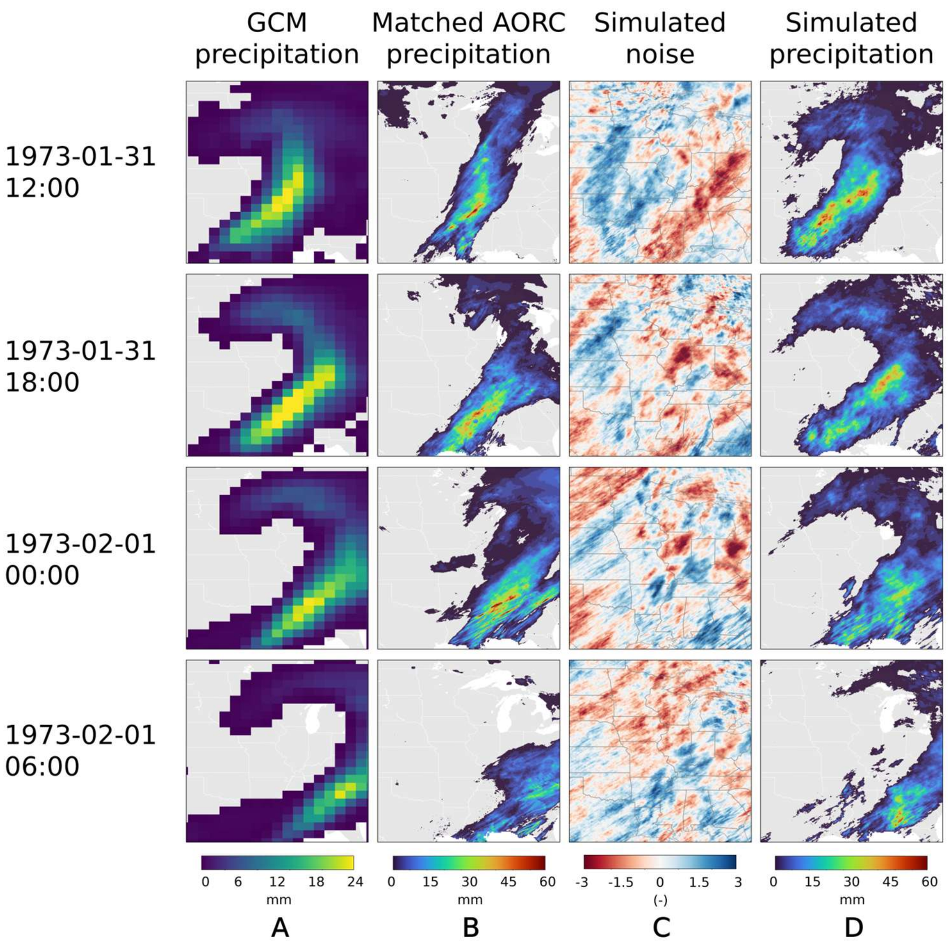

Fig. S8 Spatiotemporal patterns of a storm event starting at 12:00 UTC on 31 January 1973 from GCM data. **A,** GCM large-scale precipitation. **B,** Matched historical precipitation from the AORC dataset. **C,** Simulated Gaussian noise. **D,** Simulated high-resolution precipitation. The GCM data is from CESM2 for this example.

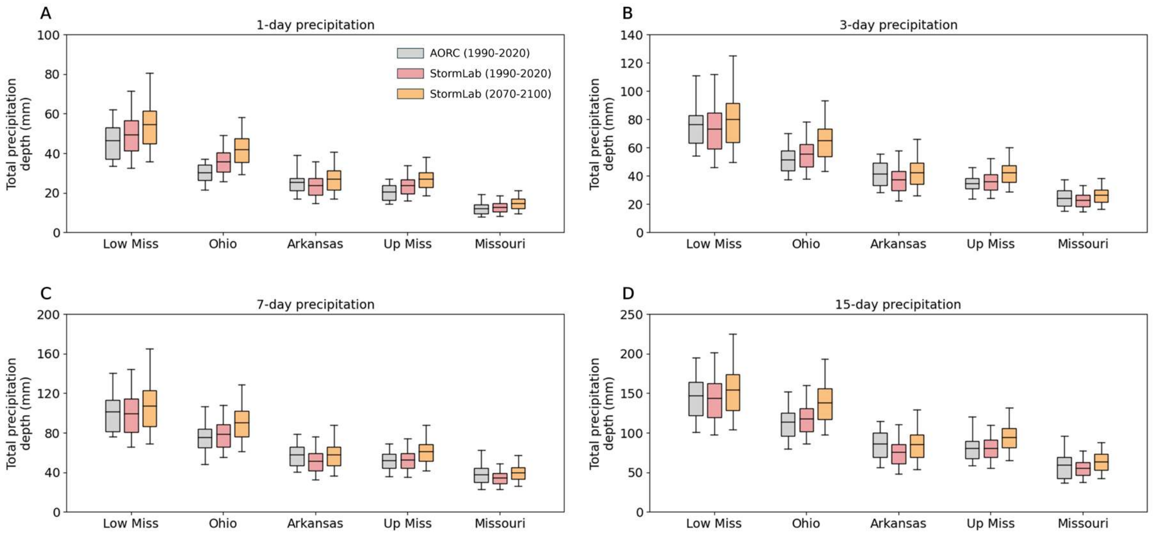

Fig. S9 Annual maximum precipitation in winter and spring seasons for the five major sub-basins. A-D, annual maxima with durations of (**A**) 1 day, (**B**) 3 days, (**C**) 7 days, and (**D**) 15 days. Gray boxes represent the AORC precipitation for 1990-2020. Red boxes represent StormLab-simulated precipitation for 1990-2020. Orange boxes represent StormLab-simulated precipitation for 2070-2100. In each plot, the box spans from the first quartile to the third quartile of the data, with a line at the median and a dot at the mean. The whiskers represent 5-95 percentiles.

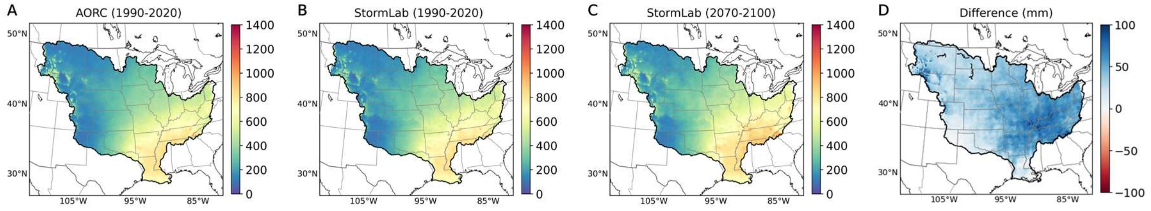

Fig. S10 Average total precipitation in winter and spring seasons in the Mississippi River Basin. A, AORC precipitation for 1990-2020. **B,** StormLab-simulated precipitation for 1990-2020. **C,** StormLab-simulated precipitation for 2070-2100. **D,** Difference between StormLab-simulated precipitation for 2070-2100 and 1990-2020.

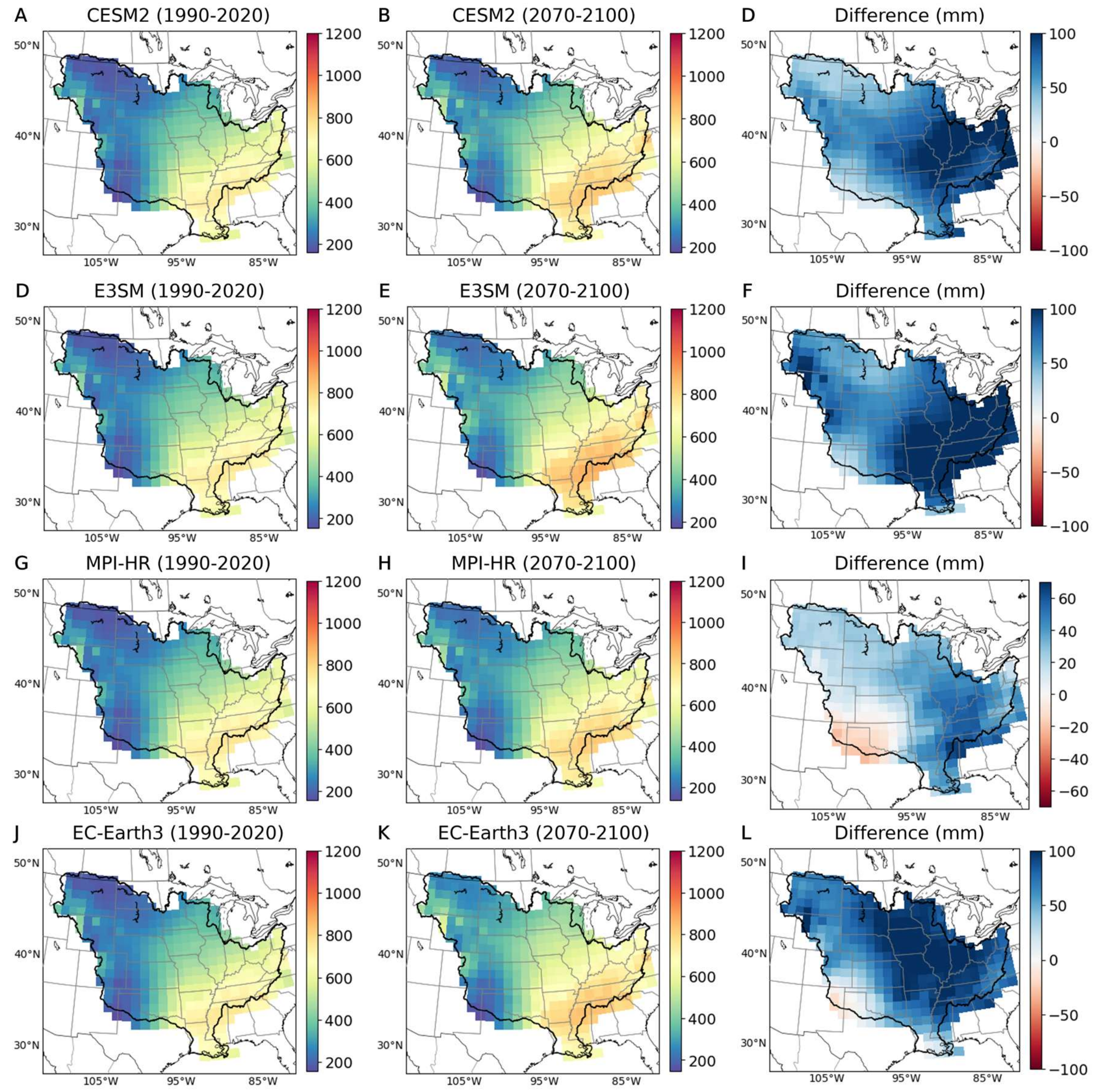

Fig. S11 Average total precipitation in winter and spring seasons in the Mississippi River Basin. A, Bias-corrected CESM2 precipitation for 1990-2020. **B,** Bias-corrected CESM2 precipitation for 2070-2100. **C,** Difference between bias-corrected CESM2 precipitation for 2070-2100 and 1990-2020. Panels (**D-L**) represent precipitation from E3SM, MPI-HR, and EC-Earth3, respectively.

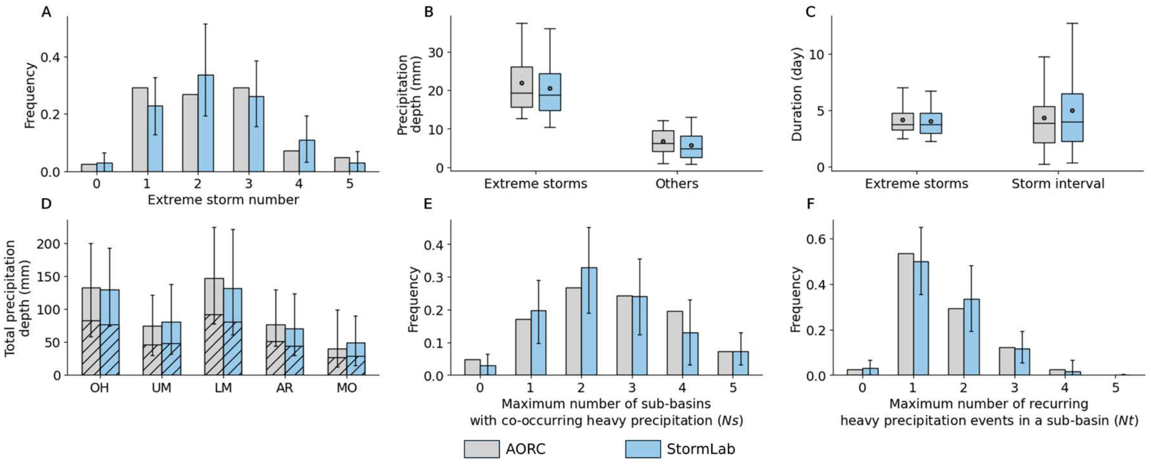

Fig. S12 Characteristics of extreme storm clusters preceding winter-spring flood peaks at Vicksburg from AORC and StormLab simulated precipitation. A, Number of extreme storms. Extreme storms are defined as events producing precipitation ≥ 90th percentile of historical records in at least one major sub-basin. **B,** Precipitation depth of extreme storms and other minor events. **C,** Extreme storm duration and dry intervals. **D,** Total precipitation depth in major sub-basins within 30 days before flood peaks. Hashed areas represent the average contribution from extreme storms. **E,** Maximum number of sub-basins affected by heavy precipitation from a single storm event. **F,** Maximum number of recurring heavy precipitation in the same sub-basin. In panels (**A**, **D-F**), bars represent the mean. In panels (**B**, **C**), boxes span the first to third quartiles, with a line at the median and a dot at the mean. Whiskers represent the 5th-95th percentiles.

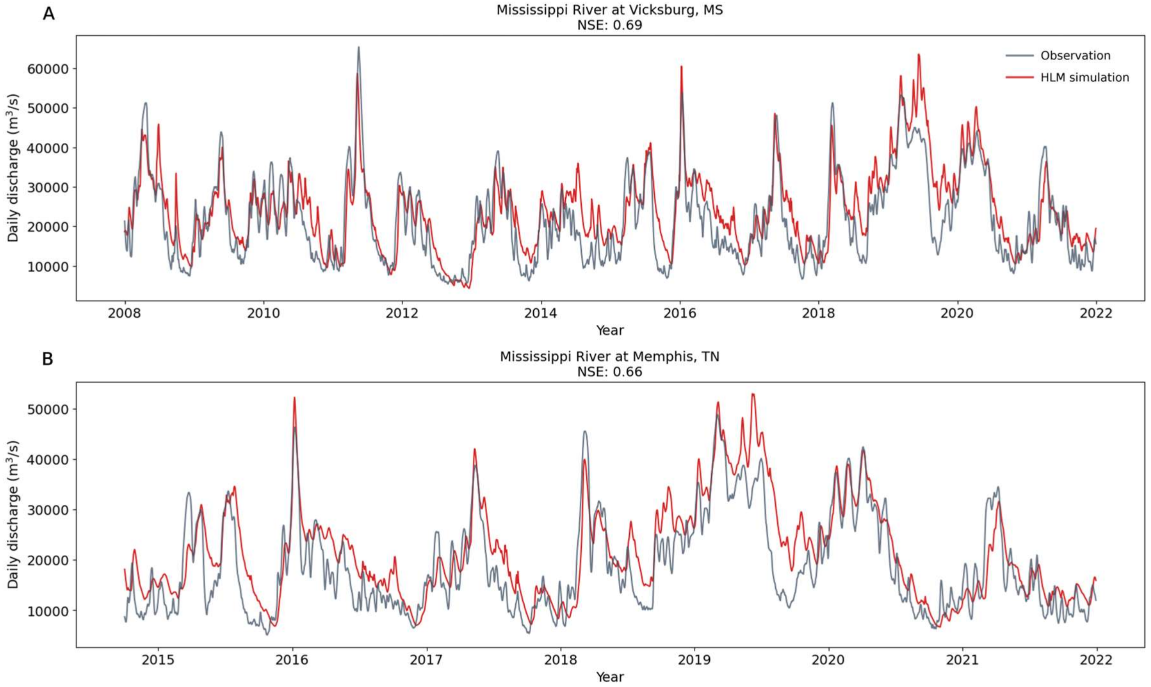

Fig. S13 Observed (gray) and simulated (red) discharge time series at Vicksburg (A) and Memphis (B) in the Lower Mississippi River Basin.

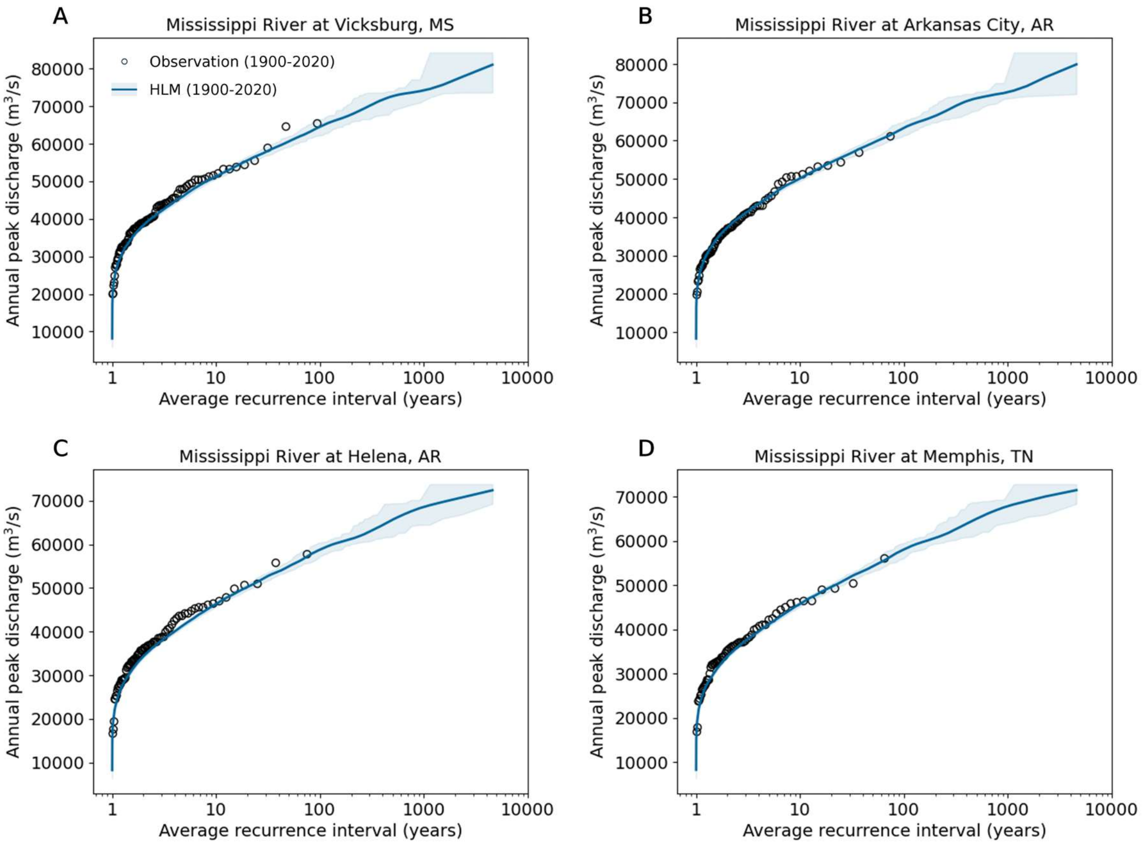

Fig. S14 Return levels of winter-spring peak discharge based on gauge observations (black dots) and Hillslope Link Model simulations (blue line) for 1900-2020. A, Vicksburg, **B,** Arkansas City, **C,** Helena, **D,** Memphis. Shaded areas represent 95% prediction intervals obtained by bootstrapping. Data are plotted using the Weibull plotting position.

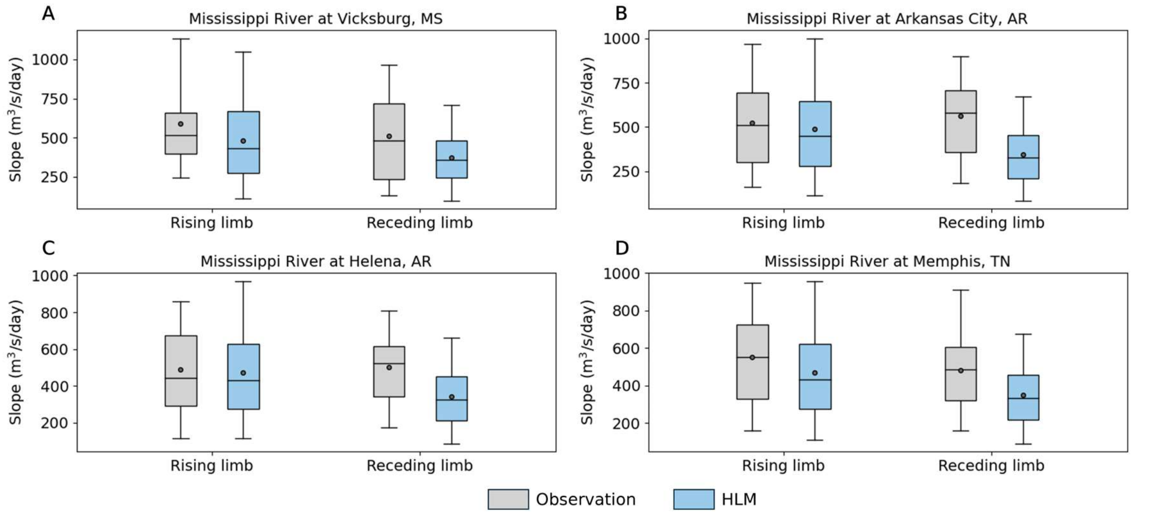

Fig. S15 Slopes of rising and receding limbs of observed and simulated flood hydrographs. A, Vicksburg, **B,** Arkansas City, **C,** Helena, **D,** Memphis. Boxes span the first to third quartiles, with a line at the median and a dot at the mean. Whiskers represent the 5th-95th percentiles.

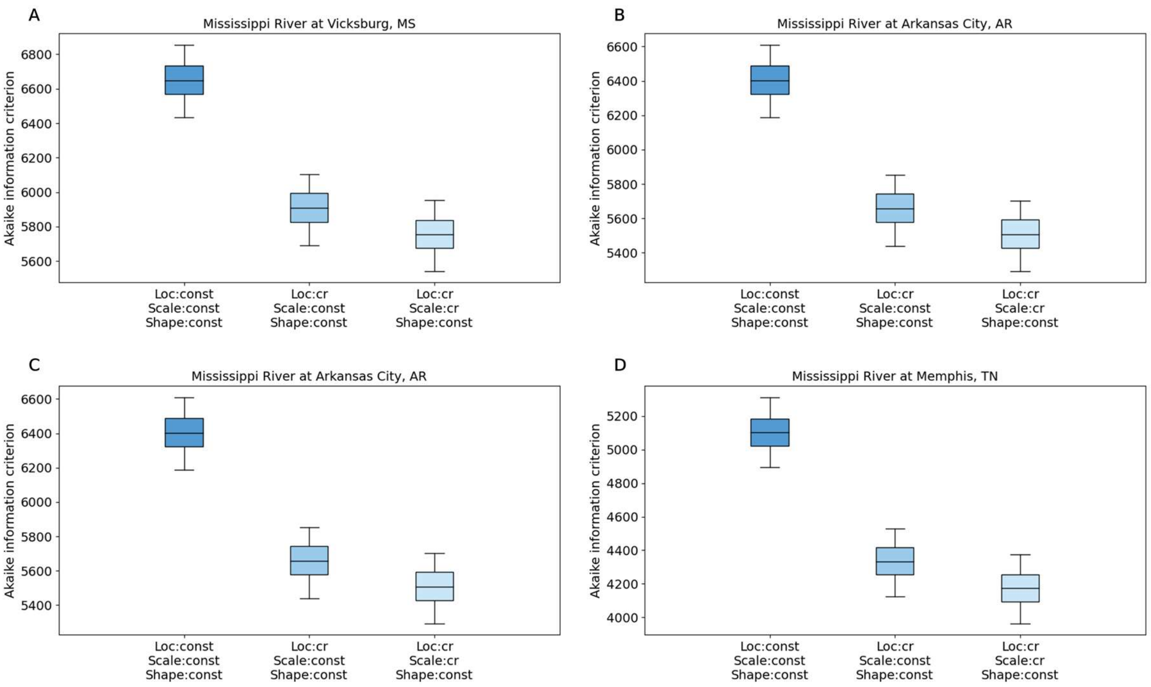

Fig. S16 Akaike information criterion of fitted GEV models. A, Vicksburg, **B,** Arkansas City, **C,** Helena, **D,** Memphis. Left box plot represents the range of Akaike Information

criterion of the stationary model, middle box plot represents the model with cubic spline regression on location parameter only, right box plot represents the model with CR on both location and scale parameters. Boxes span the first to third quartiles, with a line at the median. Whiskers represent the 5th-9th percentiles.

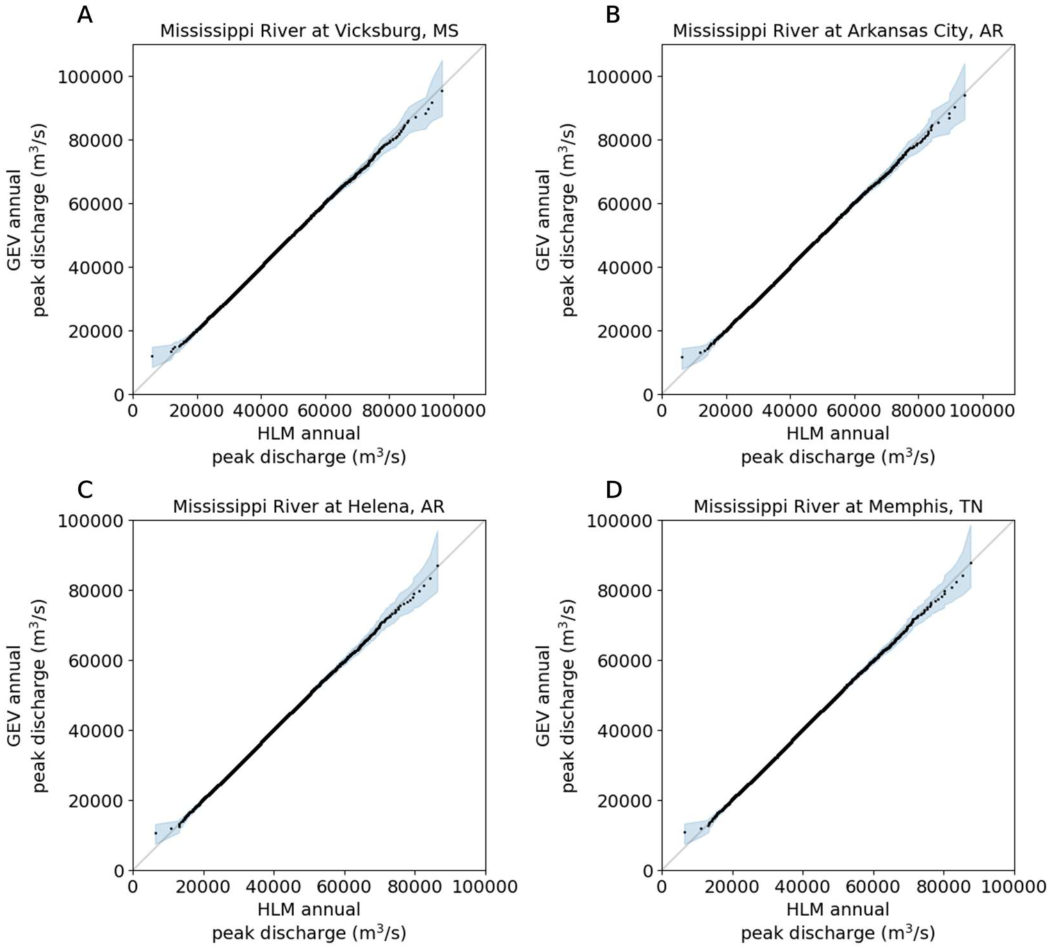

Fig. S17 Quantile-quantile plot of the nonstationary GEV distributions. A, Vicksburg, **B,** Arkansas City, **C,** Helena, **D,** Memphis. Black dots represent the median of the 1000 GEV simulations and the peak discharge simulated by the Hillslope Link Model at the corresponding quantile. Shaded areas represent 95% prediction intervals obtained by 1000 GEV simulations.

Table S1 Percentage of annual peak discharge in December-May

Location	Total number of peak observations	Annual peaks in Dec-May	Percentage (%)
Vicksburg	109	99	91
Arkansas City	87	80	92
Helena	83	80	96
Memphis	72	68	94

Table S2 Parameters used in the Hillslope Link Model in major sub-basins

Parameter	Unit	Lower Mississippi River Basin	Arkansas-Red River Basin	Ohio-Tennessee River Basin	Upper Mississippi River Basin	Missouri River Basin
Air temperature threshold	°C	0	0	0	0	0
Melting rate	mm/day/°C	2.7	2.7	2.7	2.7	2.7
Maximum storage static tank	mm	250	250	150	150	150
Upper soil infiltration rate	mm/hour	15	15	8	8	8
Overland flow velocity	mm/s	1.73	1.73	1.73	1.73	1.73
Deep soil infiltration rate	mm/hour	10	10	3	3	3
Gravitational storage residence time	days	8.11	8.11	8.11	8.11	8.11
Aquifer storage residence time	days	160	160	53	53	53
Flow velocity in channel	m/s	0.36	0.36	0.36	0.36	0.36
Discharge exponent	-	0.15	0.15	0.15	0.15	0.15
Drainage area exponent	-	-0.011	-0.011	-0.011	-0.011	-0.011

Table S3 Peak discharge of the design flood "Hypo-Flood 58A" estimated by the 1955 and 2016 study (data retrieved from (*17*)).

	1955 study			2016 study		
Location	Unregulated condition (m^3/s)	Regulated condition (m^3/s)	Difference (%)	Unregulated condition (m^3/s)	Regulated condition (m^3/s)	Difference (%)
Vicksburg	83,818	76,739	-8.5	91,888	87,103	-5.2
Arkansas City	90,897	81,836	-10.0	95,315	92,398	-3.1
Helena	76,739	69,659	-9.2	81,014	78,947	-2.6
Memphis	78,438	68,244	-13.0	83,705	81,071	-3.2

Table S4 Land use changes within the Mississippi River Basin (data retrieved from (*17*)). Special uses include rural transportation, parks and wildlife areas, defense installations, and farmsteads. Other land includes marshes, swamps, bare rock, deserts, tundra plus other uses not estimated, classified, or inventoried.

Land use	By Land Use Category			By Total Area		
	1949 (km^3)	2017 (km^3)	Difference (%)	1949, % of total areas	2007, % of total areas	Change from 1949 (%)
Cropland	1,466,204	1,286,455	-13	34%	30%	+4%
Grassland and pasture	1,284,723	1,250,268	-3	29%	29%	-1%
Forest land	1,207,890	1,250,228	3	28%	29%	-1%
Special uses	192,910	299,560	43	4%	7%	2%
Urban	38,631	111,685	97	1%	3%	2%
Other land	186,135	151,215	-21	4%	3%	-1%
